# Complementary RNA-Sequencing Based Transcriptomics and iTRAQ Proteomics Reveal the Mechanism of the Alleviation of Quinclorac Stress by Salicylic Acid in *Oryza sativa* ssp. *japonica*

**DOI:** 10.3390/ijms18091975

**Published:** 2017-09-14

**Authors:** Jian Wang, Faisal Islam, Lan Li, Meijuan Long, Chong Yang, Xiaoli Jin, Basharat Ali, Bizeng Mao, Weijun Zhou

**Affiliations:** 1 Institute of Crop Science and Zhejiang Key Laboratory of Crop Germplasm, Zhejiang University, Hangzhou 310058, China; 11216036@zju.edu.cn (J.W.); 11416096@zju.edu.cn (F.I.); 11516005@zju.edu.cn (L.L.); 3130100785@zju.edu.cn (M.L.); yangchong@zju.edu.cn (C.Y.); jinxl@zju.edu.cn (X.J.); basharat@uni-bonn.de (B.A.); 2 Institute of Crop Science and Resource Conservation (INRES), Abiotic Stress Tolerance in Crops, University of Bonn, 53115 Bonn, Germany; 3 Institute of Biotechnology, Zhejiang University, Hangzhou 310058, China

**Keywords:** *Oryza sativa* L., quinclorac, salicylic acid, RNA sequencing, iTRAQ, differentially expressed genes (DEGs), differentially expressed proteins (DEPs), transcription factor

## Abstract

To uncover the alleviation mechanism of quinclorac stress by salicylic acid (SA), leaf samples of *Oryza sativa* ssp. *Japonica* under quinclorac stress with and without SA pre-treatment were analyzed for transcriptional and proteomic profiling to determine the differentially expressed genes (DEGs) and proteins (DEPs), respectively. Results showed that quinclorac stress altered the expression of 2207 DEGs (1427 up-regulated, 780 down-regulated) and 147 DEPs (98 down-regulated, 49 up-regulated). These genes and proteins were enriched in glutathione (GSH) metabolism, porphyrin and chlorophyll metabolism, the biosynthesis of secondary metabolites, glyoxylate and dicarboxylate metabolism, and so on. It also influenced apetala2- ethylene-responsive element binding protein (AP2-EREBP) family, myeloblastosis (MYB) family and WRKY family transcription factors. After SA pre-treatment, 697 genes and 124 proteins were differentially expressed. Pathway analysis showed similar enrichments in GSH, glyoxylate and dicarboxylate metabolism. Transcription factors were distributed in basic helix-loop-helix (bHLH), MYB, Tify and WRKY families. Quantitative real-time PCR results revealed that quinclorac stress induced the expression of glutathion reductase (GR) genes (*OsGR2*, *OsGR3*), which was further pronounced by SA pre-treatment. Quinclorac stress further mediated the accumulation of acetaldehyde in rice, while SA enhanced the expression of *OsALDH2B5* and *OsALDH7* to accelerate the metabolism of herbicide quinclorac for the protection of rice. Correlation analysis between transcriptome and proteomics demonstrated that, under quinclorac stress, correlated proteins/genes were mainly involved in the inhibition of intermediate steps in the biosynthesis of chlorophyll. Other interesting proteins/genes and pathways regulated by herbicide quinclorac and modulated by SA pre-treatment were also discussed, based on the transcriptome and proteomics results.

## 1. Introduction

Rice (*Oryza sativa* L.) is one of the most important cereal crops in China, grown over an area of 30.2 million hectares and producing 208.2 million tons of rice [1]. However, weeds are one of the major biological constraints in rice production. The average yield losses in paddy field due to weeds may vary from 40% to 60%, and can even reach between 94% and 96%, depending upon the cropping system and management practices [2].

The most effective, prompt and economic method for the control of weeds is related to the use of chemicals, such as the application of herbicides. Synthetic herbicides have been used in agriculture on a global scale for about 70 years. Quinclorac (3,7-dichloro-8-quinolinecarboxylic acid) belonging to the quinoline carboxylic acid type is one of most effective synthetic herbicides, which can easily kill *Echinochloa crus-galli* (barnyard grass) and other rice weeds [3]. In China, this herbicide has been used to control barnyard grass in rice fields for almost 30 years; however, it has now evolved a resistance to quinclorac [4]. Excessive or inappropriate herbicide use to control resistant weeds can cause phytotoxicity, which may jeopardize the crop at an early stage. The concept of enhancing crop tolerance to herbicides with the use of chemical treatments was proposed in the late 1940s [5].

Modern genetic research has explicated that most genes exercise their functions through the regulation of particular proteins. Intuitively, a gene’s high level of transcripts should represent a corresponding high level of protein. However, it has been extensively demonstrated that post transcriptional processing determines steady-state protein levels [6]. RNA sequencing (RNA-Seq), as a transcript quantification technology, provides a far more precise measurement of transcripts levels and their isoforms than other approaches, and thus has been widely and successfully applied in transcript profiling, annotation and gene identification in various plant species [7,8,9]. Likewise, proteomics is also gaining recognition as a reliable and reproducible high-throughput approach for understanding biological processes [10,11]. Isobaric tagging for relative and absolute quantification (iTRAQ), a second-generation, gel-free proteomics analysis gives more accurate quantitation of protein levels.

According to our preliminary results [12,13,14], we selected SA as an antidote to alleviate quinclorac toxicity in rice plants and found that SA mainly functioned by increasing antioxidant defense and by reducing the levels of reactive oxygen species (ROS). However, the expression of genes, proteins and an understanding of the SA-mediated alleviation of quinclorac toxicity at molecular levels is still undisclosed. To provide novel insights into the molecular basis of the SA-mediated alleviation of quinclorac toxicity in rice, we therefore carried out transcriptomic and proteomic profiling to identify the changes at the level of gene expression, and post-translational modifications to elucidate the mechanisms involved in the temporal and spatial regulation of genes/proteins under SA pre-treatment and quinclorac stress.

## 2. Results

### 2.1. Primary Transcriptome Analysis

**Figure 1:**
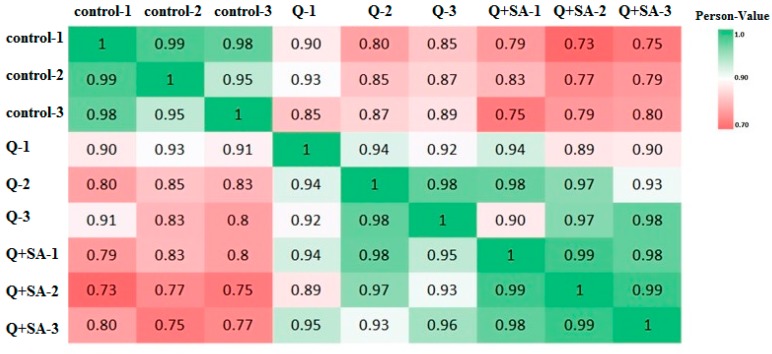
Correlation heatmap of samples. The gradient color barcode at the top-right indicates the minimum value in red and the maximum in green. If one sample is highly similar to another one, the correlation value between them is very close to 1.

In the transcriptome project, we sequenced 3 RNA-Seq groups (control, Q and Q + SA) with 3 replications, which approximately generated 13,127,032 raw sequencing reads, and then 13,046,191 clean reads after filtering low quality reads (Table 1). A control group of plants was treated with nutrient solution. The Q group of plants was treated with herbicide quinclorac (0.1 mM), whereas Q + SA treatment represents the pre-treatment of rice plants with SA (10 mg/L) for two days prior to the application of herbicide quinclorac (0.1 mM). The average mapping ratio with the reference gene is 89% and the average genome mapping ratio is 86%. We calculated the correlation value between three replicated samples based on normalized expression results and drew a correlation heatmap, as shown in Figure 1. The correlation coefficient was 99% (control-1 and control-2), 98% (control-1 and control-3), and 95% (control-2 and control-3), respectively. The correlation coefficient for Q samples was 99% (Q-1 and Q-2), while it was 94% (Q + SA-1 and Q + SA-2) for Q + SA samples, respectively, which revealed a high sample repeatability. Results demonstrated that the control group (control-1, control-2 and control-3) was significantly different from the Q group (Q-1, Q-2 and Q-3), and the Q + SA group (Q + SA-1, Q + SA-2 and Q + SA-3).

### 2.2. Protein Identification and Quantitation

**Figure 2:**
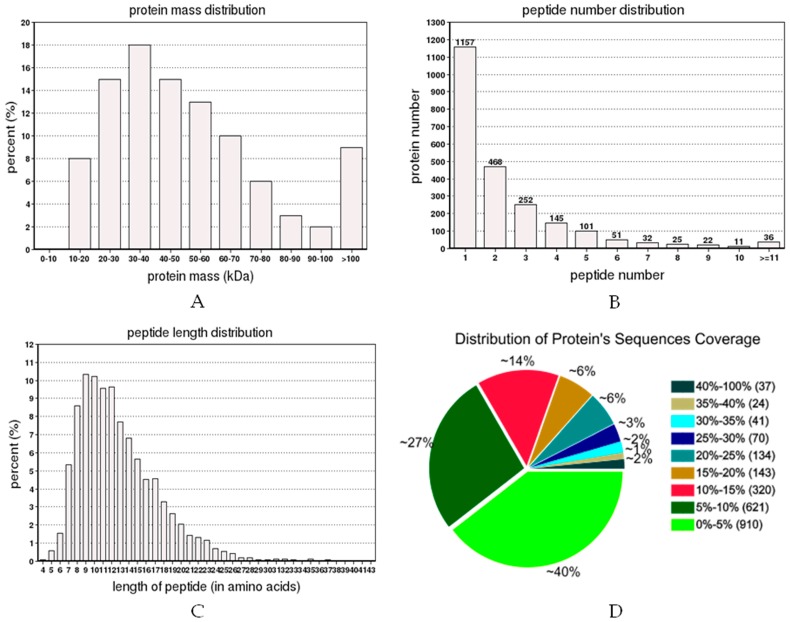
Identification and analysis of the proteome of *O. sativa* plants. (**A**) Identified proteins were grouped based on their protein mass; (**B**) Number of peptides that match to proteins as shown by Protein Pilot 5.0; (**C**) The percentage of different peptide lengths in total amino acids. (**D**) Distribution of a protein’s sequence coverage.

**Figure 3:**
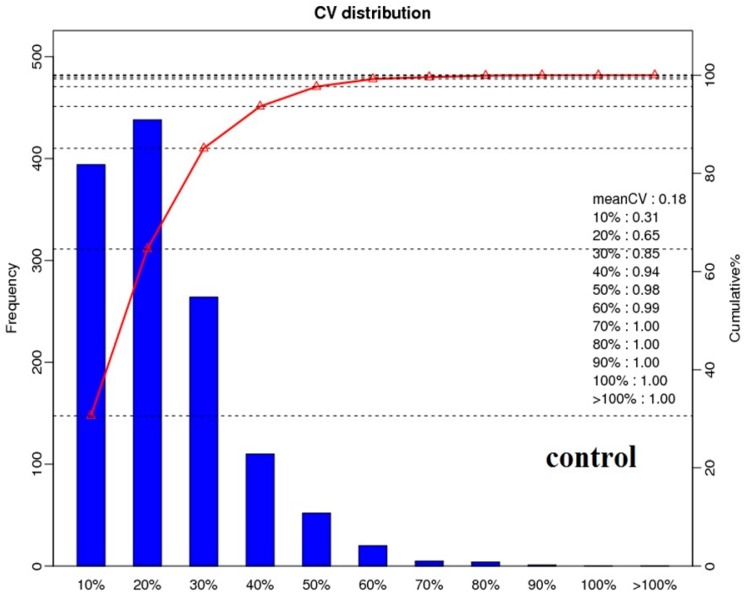

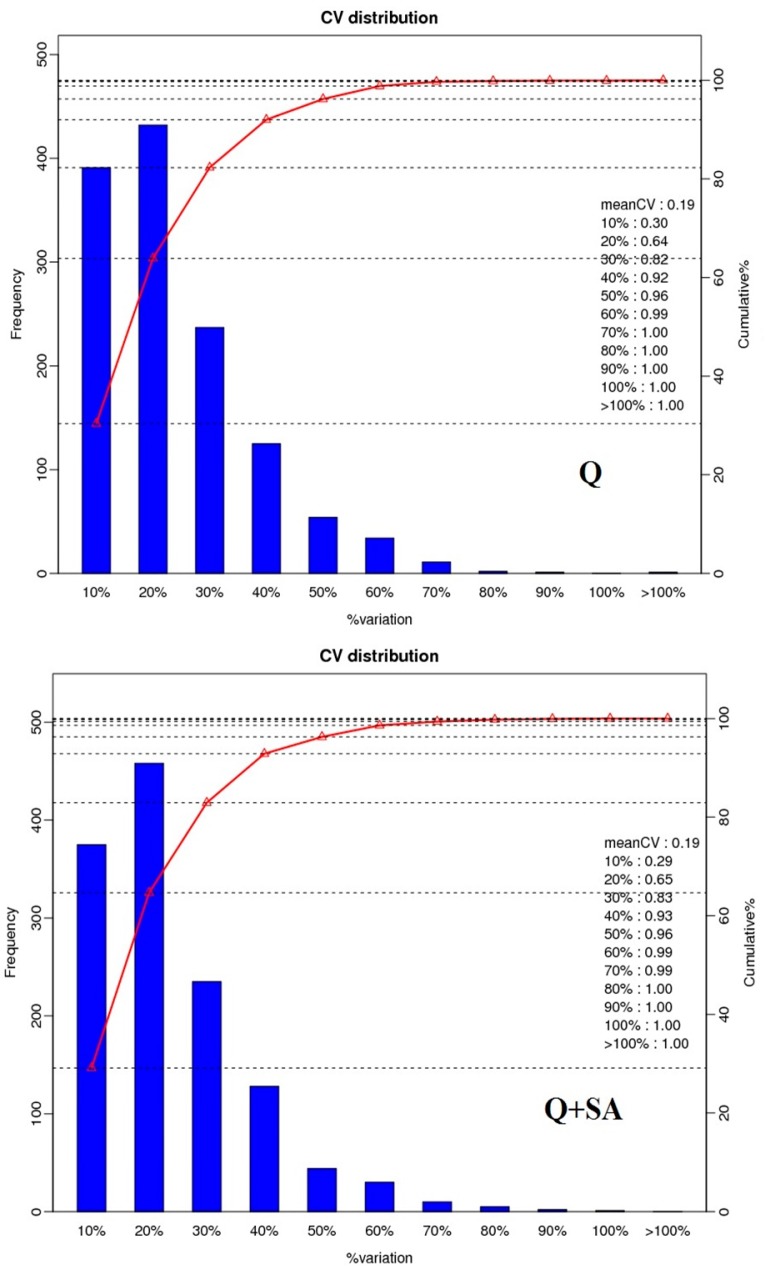
Three plots, one for each group (control, Q and Q + SA), that show the CV values (%) for comparing protein quantifications over the length of identified peptides.

A total of 291,605 spectra were produced from the iTRAQ experiment for different sets of treatments. By analyzing these spectra, we identified 17,872 known spectra, 16,184 unique spectra, 5812 peptides, 5453 unique peptides and 2300 proteins, respectively. The distribution of protein mass was summarized in Figure 2A. Proteins with 30–40 kDa accounted for 18%, followed by proteins with 20–30 kDa and 40–50 kDa. Figure 2B showed the numbers of peptide identified into the proteins. Results showed that identified proteins contained less than 10 peptides, and protein quantity decreased with the increase of the peptide. The distribution of peptide length has been shown in Figure 2C. Most of the peptides′ lengths were around nine, and the number of peptide lengths of more than 13 was considered to be relatively low. Additionally, 98% of protein sequence coverage was below 40% (Figure 2D). Meanwhile, the repetitive analysis for proteins is depicted in Figure 3. The results revealed that the CV value of three treatments (control, Q, Q + SA) was less than 0.2. The proportion of variation level between 10%–30% accounted for the major part of the total quantitative protein, which demonstrated the high repetition of each treatment.

### 2.3. Screening of DEGs and DEPs

**Figure 4:**
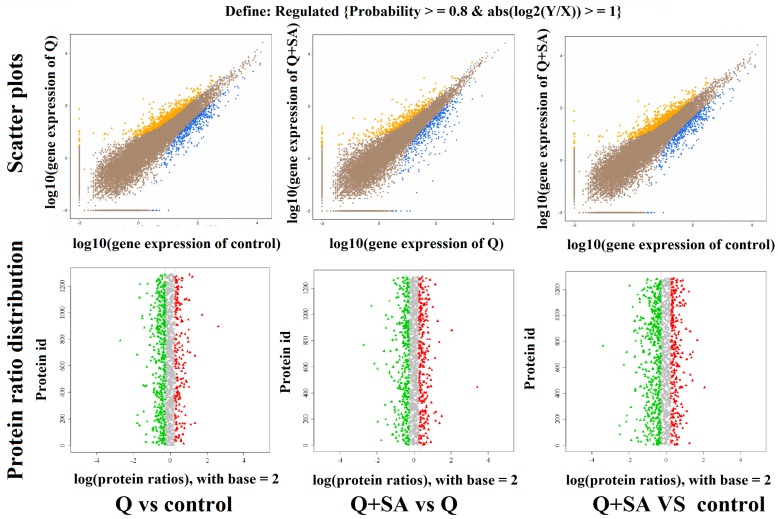
Scatter plots of gene expression level and the protein abundance distribution of expressed proteins. In the scatter plot, blue dots represent down-regulated genes, orange dots represent up-regulated genes, and brown dots represent non-regulated genes; whereas, in the protein-abundance distribution figures, green and red dots represent significantly down-regulated and up-regulated proteins, respectively. The grey dots represent insignificant changed proteins.

**Figure 5:**
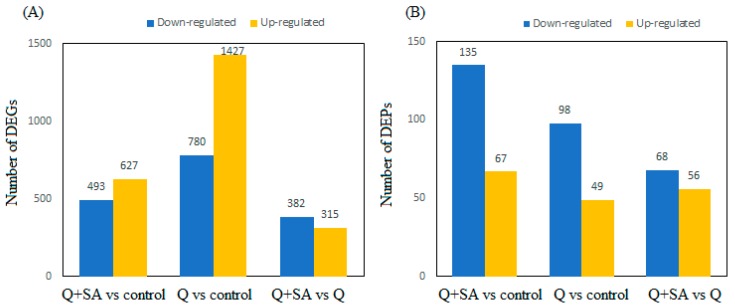
The numbers of differentially expressed genes (DEG, **A**) and proteins (DEP, **B**). The blue bar denotes down-regulated genes or proteins, and the orange bar shows the up-regulated ones.

In order to find DEGs in different transcripts, we performed function analysis via the NOISeq, a differential expression algorithm. For the NOISeq method, samples were firstly grouped so a comparison could be performed between every two groups in a pairwise manner. We drew the scatter plot of all expressed genes and protein abundance and distribution, as in Figure 4, to represent the distribution of DEGs and DEPs in screening threshold dimensions. A histogram represented the significant up/down regulation of gene and protein numbers in Figure 5. More genes were expressed under quinclorac stress, including 1427 up-regulated and 780 down-regulated, compared to the control (Figure 5A, Q vs. control). Analysis of the Q + SA vs. Q comparison showed that 315 transcripts were up-regulated, while 382 genes were down-regulated. Exogenous SA pre-treatment up-regulated 627 genes, while 493 were down-regulated. For DEPs, quinclorac inhibited 98 proteins and increased the expression of 49 proteins (Figure 5B, Q vs. control). Moreover, SA pre-treatment stimulated a similar number of proteins including 68 down-regulated proteins and 56 up-regulated proteins (Figure 5B, Q + SA vs. Q).

### 2.4. GO Functional Classification and Pathway Enrichment Analysis of DEGs

We used Web Gene Ontology (WEGO) software for a Gene Ontology (GO) functional classification to understand the distribution of genes at macro level (Figure S1). Among three comparisons, major processes were “cellular process” and “metabolic process” in the biological process category, “cell” and “cell part” categories were predominant within the cellular component category, and most genes were annotated in the “binding” and “catalytic activity” categories of molecular function (Figure S1). Additionally, we found many genes that were related to “response to stimulus” and “single-organism process” in the biological process area, “membrane” and “organelle” in the cellular component, “transporter activity” and “nucleic acid binding transcription factor activity” in the area of molecular function, whereas few genes were classified into the “positive regulation of biological process”, “cell junction” and “metallochaperone activity” groups as shown in Figure S1. Pathway enrichment analyses of the DEGs based on the Kyoto Encyclopedia of Genes and Genomes (KEGG) database were performed to study the gene interaction with each other in order to play roles in certain biological functions. The whole report for DEGs in each pairwise, respectively, was generated in Table S1. In addition, we drew a scatter plot for the top 20 KEGG enrichment results in Figure S2. Most genes were clustered in metabolic pathways and the biosynthesis of secondary metabolites. The expressions of DEGs related to the ribosome were high in the Q + SA group compared to the control. In comparison with the control, herbicide treatment caused the enrichment of most genes in glutathione metabolism, porphyrin and chlorophyll metabolism, and valine, leucine and isoleucine degradation pathways (Figure S2, Q vs. control). Genes related to glutathione metabolism were also enriched in the Q + SA vs. Q comparison. In addition, SA pre-treatment also modulated phenylpropanoid biosynthesis, tyrosine metabolism and flavonoid biosynthesis. The Q + SA vs. control comparison did not show a similar significant level to the other two comparisons (Q vs. control, Q + SA vs. Q). The main pathways, such as porphyrin and chlorophyll metabolism, carbon fixation in photosynthetic organisms and photosynthesis, played an important role in the comparison. Over the total 16811 annotated genes, quinclorac produced 1289 genes enriched in 121 pathways, while 40 pathways were significantly enriched (*p* ≤ 0.05) (Table S1.1). SA with quinclorac stress generated 407 genes enriched in 108 pathways, while 31 pathways were significantly enriched (Table S1.2). In the Q + SA vs. control comparison, a total of 649 genes were enriched in 102 pathways with only 16 significant enrichments (Table S1.3).

Within the top five most significantly enriched pathways, glutathione metabolism and biosynthesis of secondary metabolites were found to be the most-enriched pathways under quinclorac stress, which proved that glutathione and secondary metabolites play important roles in herbicide detoxification. Under quinclorac stress, a total of 46 DEGs related to the glutathione pathway were expressed, while 260 DEGs related to secondary metabolites were expressed, whereas SA pre-treatment regulated 27 and 97 DEGs in these two pathways, accounting for 7% and 24% of all expressed DEGs, respectively. In the Q + SA vs. control comparison, DEGs related to ribosomal synthesis/regulation were enriched. Besides this, porphyrin and chlorophyll metabolism, carbon fixation in photosynthetic organisms, aminoacyl-tRNA biosynthesis pathways were also dominated pathways among other expressed pathways.

### 2.5. Overview of Metabolism by Using MapMan

**Figure 6:**
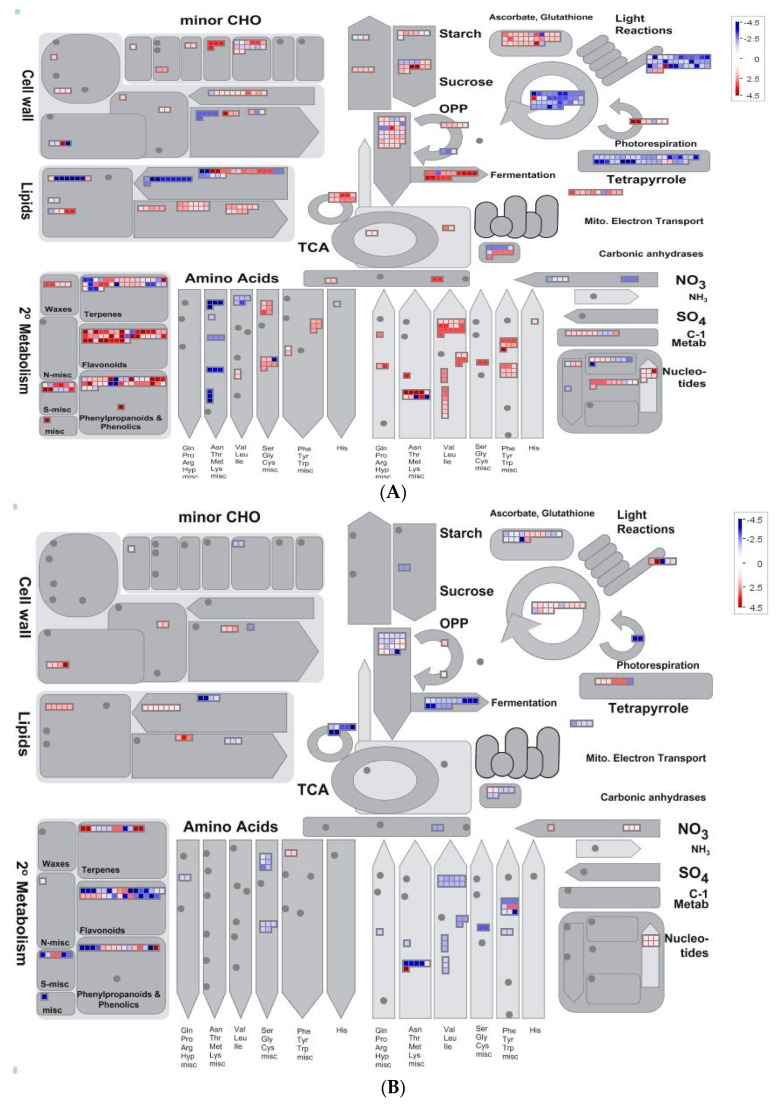

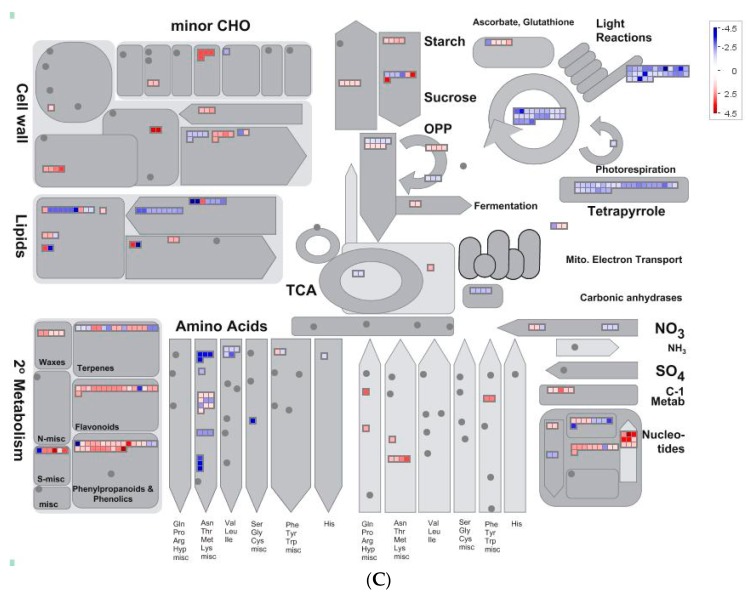
Overview of differentially expressed genes involved in various metabolic processes under different treatments. (**A**) Q vs. control; (**B**) Q + SA vs. Q; and (**C**) Q + SA vs. control. The images were obtained using MapMan, showing different functional categories that passed the cutoff (less than 0.05 q value and greater than two-fold change) for differential expression. The red color represents up-regulated genes, and the blue color represents down-regulated genes.

We downloaded MapMan software from the Genome Analysis of the Plant Biological System (GABI) Primary Database and made a visual overview of metabolism (Figure 6). As shown in Figure 6A–C, quinclorac induced genes related to ascorbate and glutathione metabolism and inhibited light reaction and photorespiration to inhibit photosynthesis. The secondary metabolism contained a number of genes participating in the synthesis of flavonoids, phenylpropanoids and phenolics. Genes in the cell wall, lipids, amino acids and starch metabolism varied differently; some were up-regulated and some were down-regulated (Figure 6A). SA further stimulated the expression of ascorbate and glutathione genes. Light reaction and photorespiration were enhanced, and genes in the cell wall and lipid metabolism were increased (Figure 6B). In the Q + SA vs. control comparison, genes related to secondary metabolism were abundantly expressed, particularly in relation to the flavonoids, phenylpropanoids and phenolic metabolism (Figure 6C).

### 2.6. Quantitative PCR Analysis of Candidate Genes

**Figure 7:**
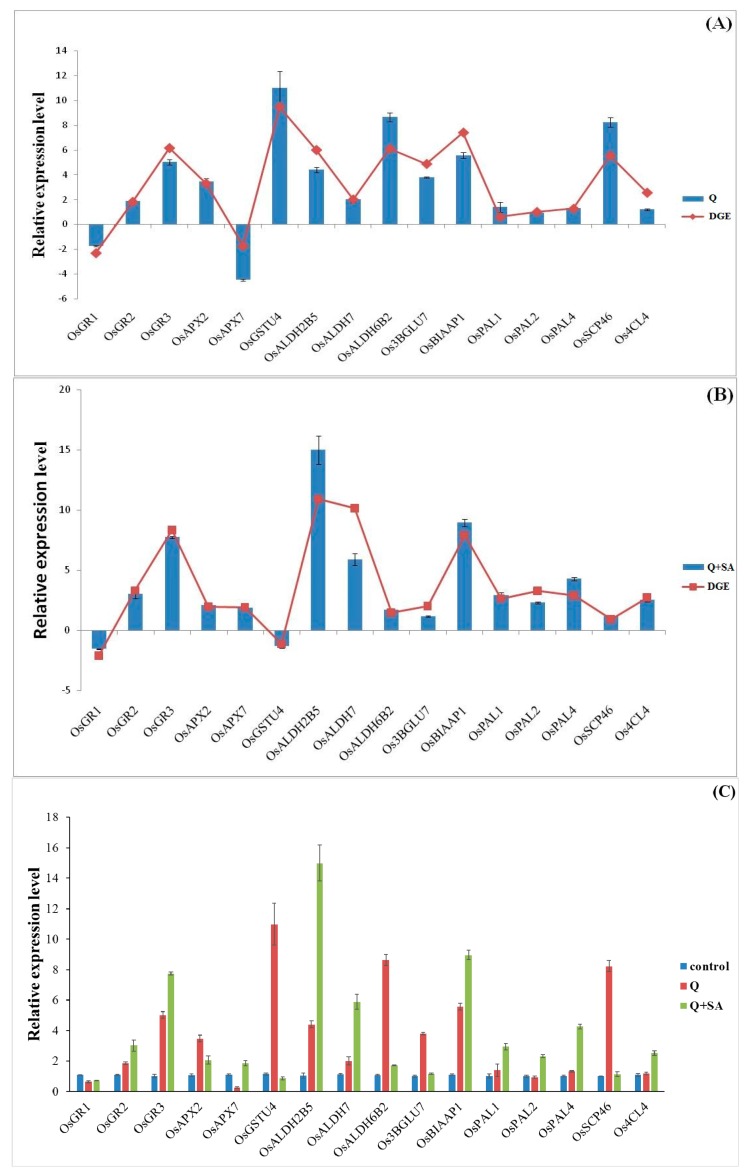
Confirmation of expression levels of selected differentially expressed genes (DEGs) by quantitative real time-PCR assays. (**A**) herbicide quinclorac treatment; (**B**) salicylic acid pre-treatment under quinclorac stress; and (**C**) various genes expression under different treatments. The data show the averages and the standard deviation of three independent samples.

According to the pathway enrichment analysis of DEGs, we randomly selected some candidate genes from significantly enriched pathways to verify the RNA-Seq data (Figure 7). Moreover, real time-PCR results also confirmed our RNA-Seq data. Glutathione reductase (GR) genes (*OsGR2*, *OsGR3*) were largely up-regulated under quinclorac stress, while the expressions of said genes were further enhanced under SA pre-treatment. The expression of *OsGSTU4* and *Os3BGLU7*, which determines glutathione S-transferase (GST) and β-glucosidase respectively, were quite high after quinclorac treatment, while SA had no significant effect on them. Other rice acetaldehyde dehydrogenase (ALDH) genes such as *OsALDH2B5*, *OsALDH7* were also increased under quinclorac stress, and SA pre-treatment further enhanced their expressions. Moreover, SA induced phenylalanine deaminase genes (*OsPAL1*, *OsPAL2*, *OsPAL4*) and 4-coumarate: coenzyme A ligase gene (*Os4CL4*) were highly expressed (Figure 7).

### 2.7. Pathway Enrichment Analysis of DEPs

The significantly enriched pathways (*p* ≤ 0.05) under different treatments are presented in Table 2. Under quinclorac application, 87% of DEPs were enriched in seven pathways. The cysteine and methionine metabolism, glyoxylate and dicarboxylate metabolism, and phenylpropanoid biosynthesis were the most remarkable pathways, while metabolic pathways had the largest number of DEPs (Table 2, Q vs. control). On the other hand, SA + Q treatment regulated 20 proteins in five pathways, including alanine, aspartate and glutamate metabolism, riboflavin metabolism, arginine biosynthesis, terpenoid backbone biosynthesis, and 2-Oxocarboxylic acid metabolism. In Q + SA vs. control, DEPs were involved in the biosynthesis of antenna proteins, amino acids, valine, leucine and isoleucine, and the metabolism of pyrimidine, pentose and glucuronate. In a comprehensive perspective, cysteine is the product of methionine metabolism and an important part of GSH. Simultaneously, it is a crucial component of the DNA-binding domain. The enrichment of proteins in different pathways was consistent with the transcriptome results.

### 2.8. Transcription Factor Analysis

The transcription factor (TF) is also called the trans-acting factor, a kind of protein which can uniquely bind genes of 5′ upstream specific sequences, thereby ensuring target gene expression with a given rate in a particular time and space. We used hmmsearch to search for the characteristics of the domain in PlantTFDB database (http://plntfdb.bio.uni-potsdam.de/v3.0/), then conversely to predict if a gene can code TF. Meanwhile, TF was aligned from China Rice Data Center (http://www.ricedata.cn/gene/).

In the Q vs. control comparison, we found 14 TF families, mainly distributed in AP2-EREBP, MYB, and WRKY TFs of AP2-EREBP transcription families; the WRKY family were mostly up-regulated, but the MYB family showed mixed expression. It indicated that, under quinclorac stress alone, most genes were up-regulated (Table S2.1). *OsDREB2A* (LOC_Os01g07120), *OsERF922* (LOC_Os01g54890), *OsEREBP1* (LOC_Os02g54160) genes were expressed 5.16, 16.35, 2.81 times higher than control. *OsPHR3* (LOC_Os02g04640) gene belonging to MYB family was up-regulated more than tenfold. Some TFs from the no apical meristem (NAM), ATAF, and cup-shaped cotyledon (CUC) transcription factor amily, *OsNAC10* (LOC_Os11g03300) and *ONAC131* (LOC_Os12g03040) were expressed up to 62.64 and 125 times higher, respectively. In the Q + SA vs. Q comparison, we found nine TF families, and SA pre-treatment mainly repressed NAC family TFs (Table S2.2). *OsIRO2* (LOC_Os01g72370) gene, an Fe-deficiency-inducible Bhlh family TF involved in Fe homeostasis in rice, was expressed nearly 30 times. *ONAC095* (LOC_Os06g51070) gene was found to be up-regulated while the expression of *OsNAC10* (LOC_Os11g03300) and *ONAC131* (LOC_Os12g03040) genes were inhibited. Some WRKY family TFs, including *OsWRKY45* (LOC_Os05g25770) and *OsWRKY76* (LOC_Os09g25060), showed decreased transcript abundance. In the SA + Q vs. control comparison, there were also 14 TF families responding the treatment (Table S2.3). Most of TFs were found to be up-regulated, however only MYB-related TFs were inhibited.

### 2.9. Correlation Analysis of Transcriptome and Proteome Data under Different Treatments

An expression correlation analysis was performed between DEPs and their corresponding transcripts (Table 3). Detailed correlation analysis results have been shown in supplementary data (Table S3). In total, 44 correlated proteins were observed under quinclorac treatment alone; however, exogenous SA pre-treatment only generated five proteins (Table 3). In the Q + SA vs. control, we detected 35 correlated proteins. Among those proteins, 30 proteins which included four up-regulated and 26 down-regulated proteins exhibited the same expression tendency. Proteins which are related to the synthesis of chlorophyll such as Mg-chelatase H subunit (*OsChlH*, LOC_Os03g20700), catalase (CAT) (*OsCATA*, LOC_Os02g02400), geranylgeranyl reductase (*LYL1*, LOC_Os02g51080) and protochlorophyllide oxidoreductase B (*OsPORB/FGL*, LOC_Os10g35370) were inhibited. Under quinclorac stress, the synthesis of chlorophyll-related DEGs/DEPs were considerably down-regulated. Only cytosolic pyruvate kinase (*OsPK1*, LOC_Os11g05110), indole-3-glycerol phosphate synthase family protein (LOC_Os08g23150.1|PACid: 24101120), glucose-6-phosphate dehydrogenase 4 (LOC_Os03g20300.1|PACid: 24121754), lactate/malate dehydrogenase 1 (LOC_Os12g43630.1|PACid: 24149304) were expressed at differential levels (Table S3). SA pre-treatment had less significantly correlated proteins including phenylalanine ammonia-lyase (*OsPAL1*, LOC_Os02g41630), rice ferritin (*OsFER2*, LOC_Os12g01530), manganese superoxide dismutase 1 (LOC_Os05g25850.1|PACid: 24151755), phosphoenolpyruvate carboxylase family protein (LOC_Os12g08760.1|PACid: 24145205), eukaryotic aspartyl protease family protein (LOC_Os12g39360.1|PACid: 24145239) (Table S3). In the Q + SA vs. control, there were 27 correlated proteins in the same trend, and only rice heat shock protein 70 (LOC_Os03g16860.1|PACid: 24123810) was up-regulated (Table S3). In general, the correlated proteins and genes mostly showed a down-regulation trend.

### 2.10. Functional Networks of the Selected Differentially Regulated Proteins

**Figure 8:**
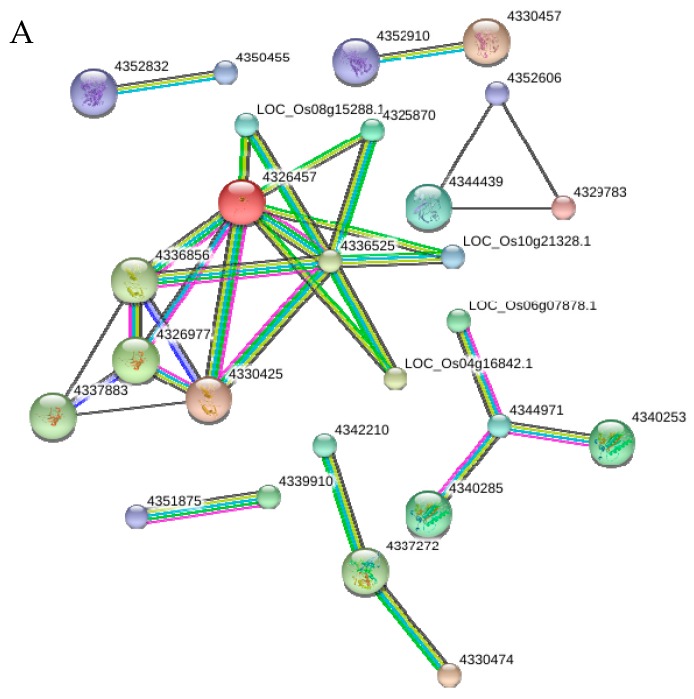

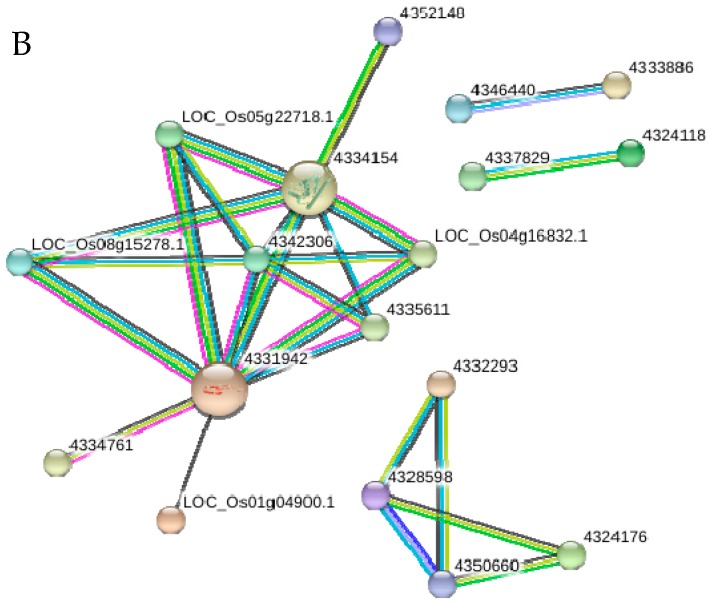
STRING software predicted differentially accumulated proteins association between herbicide quinclorac-treated only and salicylic acid + quinclorac treatment: (**A**) down-regulated and (**B**) up-regulated proteins. Differentially accumulated proteins are represented by node, whereas different color of lines represents different evidences for the predicted functional relationship between proteins; red line: gene fusion evidence; dark blue line: co-occurrence evidence; ml black line: co-expression evidence; yellow line: text-mining evidence; green line: neighborhood genome evidence; light blue line: database evidence; and pink line: experimental evidence. All interactions had a confidence score ≥0.9 (highest confidence) to minimize false positives/negatives.

A Search Tool for the Retrieval of Interacting Genes/Proteins (STRING) database of protein interaction was used to reveal a putative protein association network between herbicide quinclorac-treated alone and SA + quinclorac treatment (Figure 8). The nodes represent the proteins, and the line colors between the nodes indicate protein-protein interaction modes (Figure 8). Under down-regulated proteins, a larger participated protein interaction was found in the ribosomal proteins and RNA polymerase subunits, while other interactions involving photosynthesis and peptidase and glutamine-related proteins had a higher level of co-expression (Figure 8A). Under up-regulated proteins, higher co-expression between ribosomal proteins such as ribosomal protein L13 (LOC_Os03g54890.1), S10/S20 domain containing ribosomal protein (LOC_Os03g10060.1; LOC_Os08g15278.1), eukaryotic translation initiation factor 3 (LOC_Os07g03230.1; LOC_Os04g16832.1), S1 RNA binding domain-containing protein (LOC_Os03g62780.1) and chloroplast ribosomal proteins such as chloroplast 30S ribosomal protein S8 (LOC_Os04g16832.1; LOC_Os05g22718.1) were found. These interacted proteins are involved in tRNA binding and also operated as a transcriptional elongation factor to initiate protein synthesis. In addition to this, the strong co-expression of predicted protein-protein interaction (PPI) revealed strong association between proteins involved in regulation of the citric acid cycle, which includes dehydrogenase (LOC_Os01g46610.1), citrate synthase (LOC_Os02g10070.1), citrate synthase, putative (LOC_Os11g33240.1), and phosphoenolpyruvate carboxykinase (LOC_Os03g15050.1) protein (Figure 8B).

## 3. Discussion

In our previous experiment, we found that quinclorac considerably arrested rice growth, while SA pre-treatment alleviated quinclorac toxicity effectively [12]. Therefore, to find the changes at transcription/translation levels, we performed RNA-Seq and iTRAQ analyses respectively to investigate the changes in the transcriptome and proteome of rice leaves under quinclorac alone and SA pre-treated quinclorac stressed plants at an early stage (6 h). Under quinclorac stress, there were 2207 DEGs (1427 up-regulated, 780 down-regulated), which were enriched in the metabolic processes of glutathione, porphyrin and chlorophyll, amino acid, ribosome, glyoxylate and carbon fixation. TFs were primarily distributed in AP2-EREBP, MYB, WRKY family. However, SA + Q treatment induced 697 genes and inhibited 382 genes. The pathway analysis of these genes showed that the metabolism of glutathione, phenylpropane, tyrosine, flavonoid, lipids and glyoxylate were significantly enriched pathways. The TF family contained bHLH, MYB, Tify, WRKY and other classes of TF. Proteomic data showed that quinclorac stress induced 49 DEPs, and inhibited the expression of 98 proteins. Pathway enrichment analysis showed that these proteins were related to cysteine and methionine metabolism, glyoxylate and dicarboxylate metabolism and phenylpropanoid biosynthesis. The pre-treatment of SA induced the expression of 56 proteins, while 68 proteins were down-regulated. The most abundant pathways were alanine, aspartate and glutamate metabolism, riboflavin metabolism, arginine biosynthesis, terpenoid backbone biosynthesis, and 2-oxocarboxylic acid metabolism. Correlation analysis between transcriptome and proteomics exhibited that, under quinclorac stress, proteins related to the synthesis of chlorophyll such as Mg-chelatase H subunit, CAT, geranylgeranyl reductase and protochlorophyllide oxidoreductase B were inhibited, whereas SA regulated the expression of phenylalanine ammonia-lyase, rice ferritin and Mn-SOD family proteins.

Glutathione metabolism plays an important role in plant abiotic stress response. Glutathione is a tripeptide with γ-glutamic acid, cysteine and glycine, which is effective in eliminating ROS and exotic hazardous compounds and metabolites [15]. GR, as the only electron donator for using nicotinamide adenine dinucleotide phosphate (NADPH), can catalyze oxidized glutathione (GSSG) to reduced GSH to maintain high GSH/GSSG ratio in the cell [16]. In our study, two important genes (*OsGR2* and *OsGR3*) encoding GR were strongly expressed. Kaminaka et al. [17] and Hong et al. [18] found *OsGR2* and mRNA mainly exist in root and callus. Abscisic acid (ABA), low temperature, drought, salinity and other stresses can induce significant expression of *OsGR2* [16]. Another gene *OsGR3* is primarily expressed in roots at seeding stage and ubiquitously expressed in all tissues except the sheath at heading stage [19]. The results of qPCR demonstrated that the expression of *OsGR2* and *OsGR3* was higher in SA pre-treatment than in quinclorac alone treatment. Moreover, Dat et al. [20] also demonstrated that SA can elevate the activity of GR in *Brassica juncea*.

Endogenous aldehyde is a common metabolic intermediate produced from a number of pathways, including the metabolism of amino acids, protein, lipids and carbohydrates [21], meanwhile xenobiotics are important sources of aldehydes production [22]. ALDH is also a common and important detoxifying enzyme in plant tissues, which is responsible for eliminating endogenous and exogenous aldehydes [23]. At present, a number of studies have revealed that *ALDH* genes can be induced under salinity and drought stress, suggesting possible roles in improving abiotic stress tolerance [24,25]. Under flooding stress, *OsALDH2B5* is induced to stimulate the synthesis of ABA [26]. Further, Wu et al. [27] found that *OsALDH7* was induced by biotic or abiotic factors such as ultraviolet, blast and mechanical injury in rice leaves. Exogenous plant growth regulators treatment, such as SA, ABA and methyl ester of jasmonic acid (JA) induced the expression of *OsALDH7*. *OsALDH2B5* is located in the mitochondrion and *OsALDH7* is located in the cytoplasm [28,29]. In our present study, three *ALDH* genes (*OsALDH2B5*, *OsALDH6B2*, *OsALDH7*) were significantly expressed under quinclorac stress, whereas exogenous SA pre-treatment further increased the expression of *OsALDH2B5* and *OsALDH7*. The proteomics data also showed that quinclorac induced glycolate oxidase (GLO), which is a crucial enzyme in photorespiration and catalyzes the oxidation of glycolate to glyoxylate, with an equimolar amount of H_2_O_2_ production [30]. Noctor et al. [31] found nearly 70% H_2_O_2_ content in C3 plants coming from photorespiration via GLO catalysis. Moreover, GLO has been observed in response to abiotic or biotic stresses, such as drought and pathogens [32,33]. In our study, the GLO was significantly induced under quinclorac stress, leading to the overproduction of H_2_O_2_ that may inhibit the activity of CAT. Zhang et al. [34] also demonstrated that GLO physically interacts with CAT in rice leaves, and the interaction can be down-regulated by SA. *OsSCP46* is a serine carboxypeptidase gene and can be induced by ABA and inhibited by brassinolide [35]. Quinclorac improved *OsSCP46* gene expression, which may contribute to overproduction of ABA. 4-Coumarate: coenzyme a ligase is a vital enzyme in the phenylpropanoid metabolic pathways for monolignol and flavonoid biosynthesis. Rice genome has five *Os4CL* genes without tissue specific expression but with apparent differences in expression levels. The rank in order of transcript abundance was as *Os4CL3 > Os4CL5 > Os4CL1 > Os4CL4 > Os4CL2* [36]. SA may influence *Os4CL* gene expressions to adjust the synthesis of lignin and flavonoid. *OsBIABP1* gene is involved in the regulation of AMP-binding protein in rice defense system. Some small molecules such as SA and JA can respectively induce the expression of *OsBIABP1* to regulate the defense signaling pathways related to SA and JA/ethylene, respectively [37]. The treatments of quinclorac and SA both enhanced *OsBIABP1* gene expression, which suggests that plant responds to different types of stress factors by reprogramming the expression of similar signaling pathways.

TFs almost participate in all of the biochemical reactions and processes. AP2-EREBP TFs are known to be unique in plants and have more than 180 members in rice. According to a number of AP2-EREBP structural domains, this family can be divided into an EREBP subfamily containing one domain and AP2 subfamily containing two domains. The EREBP subfamily includes dehydration responsive element binding (DREB), ethylene responsive element binding factors (ERF) and other subfamilies. Most EREBP subfamily TFs take part in plant stress response [38,39]. Our results showed that *OsDREB2A*, *OsERF922*, *OsEREBP1* were induced to express under quinclorac stress. Over-expressed *OsDREB2A* in transgenic soybean can improve salinity stress tolerance [40]. Cui et al. [41] also observed the overexpression of *OsDREB2A* under drought and salinity stress. ABA and salinity application, as well as blast fungus, strongly induce the expression of *OsERF922*. Transgenic rice with *OsERF922* overexpression down-regulated the expression of defense genes and decreased tolerance to salinity with an increased Na^+^/K^+^ ratio [42]. When *OsEREBP1* is overexpressed, JA and ABA synthesis and signal pathways are activated to enhance drought and flooding tolerance [43]. NAC TFs, refer to new largest TF family in plants in recent years and they play important roles in the regulation of plant growth and development and participate in defense reactions of several adverse abiotic stresses including drought, high salt, low temperature [44]. Our results showed that *OsNAC10*, *ONAC131* were highly induced by quinclorac application. Jeong et al. [45] found that over-expressed *OsNAC10* significantly strengthened stress tolerance. In our experiment, we also found that quinclorac induced bHLH family gene *OsIRO2*, which has been found to be a key regulatory gene for Fe acquisition. Overexpression of *OsIRO2* can increase secretion of mugineic acid family phytosiderophores without any adverse effect on the plant [46,47]. TFs from the NAC family had inconsistent changes with SA pre-treatment, so *OsNAC095* was induced while *OsNAC10* and *ONAC131* were down-regulated. Although *OsNAC10* and *ONAC131* can be induced, this varies depending on time [48]. Huang et al. [49] found drought, salt, heat and ABA (except cold) enhanced the expression of *OsNAC095*. TFs from WRKY superfamily also take part in several physiological and resistance reactions [50,51]. Researchers have identified 109 WRKY TFs in rice [52]. *OsWRKY45* has two allelic genes (*OsWRKY45-1*, *OsWRKY45-2*), these two genes also have different transcriptional responses to ABA and salt stress [53]. *OsWRKY45* was expressed under exogenous SA pre-treatment in our study, consistent with the results of Ryu et al. [54]. The overexpression of *OsWRKY76* has been documented to suppress the induction of gene expressions involved in disease resistance and phytoalexins synthesis, leading to the increased expression of abiotic stress-associated genes such as peroxidase and lipid metabolism genes [55].

Transcriptome and proteome constantly adjust to each other at the same time when plants are under environmental stresses [56]. The correlation analysis between transcriptome and proteome showed that the majority of proteins correlated under quinclorac stress were inhibited and *OsChlH*, *OsCATA*, *LYL1*, *OsPORB/FGL* were significantly down-regulated (Table S3). *OsChlH* encodes the Mg^2+^-chelatase H subunit, which is involved in chlorophyll biosynthesis [57,58]. *LYL1* is a light responsive gene participating in the final step of chlorophyll biosynthesis and prevents the rice from lipid peroxidation and reactive oxygen damage [59]. Yang et al. [60] also found *OsPORB/FGL* mutant had phenotypically pale-green leaves with significantly decreased chlorophyll (*a* and *b*) and carotenoid contents. In addition to this, we observed that quinclorac dramatically repressed the synthesis of chlorophyll (Chl), which may be the reason of stunt growth of rice plants. In the porphyrin and chlorophyll metabolism pathway, 21 DEGs were inhibited, while only six DEGs were induced. Protoporphyrin IX, as the precursor of Chl, is synthesized by 5-aminolevulinate acid [61]. This early enzymatic reaction was suppressed by quinclorac so that the subsequent catalyzed product protoheme was simultaneously restrained. In the last step of Chl biosynthesis, prenylation of chlorophyllide was also affected, which is believed to be catalyzed by Chl synthase with phytyl diphosphate or geranygeranyl diphosphate [62,63]. The final step in converting chlorophyllide to Chl *a* or Chl *b* is taken place in the thylakoid membrane, which play important roles in the stabilization of the thylakoid membranes [64]. Recently, Wang et al. [12] observed that quinclorac application damage the thylakoid membranes, which may be due to the reduction in the biosynthesis of Chl in treated rice plants. However, pre-treatment of SA under quinclorac stress prevented the thylakoid membrane disruption by accelerating Chl production and scavenging ROS, indicating a key role in SA-induced oxidative stress tolerance in rice plants [12].

## 4. Materials and Methods

### 4.1. Plant Materials

The seeds of quinclorac tolerant *japonica* variety (*Oryza sativa* L. cv. Xiushui 134), were obtained from the College of Agriculture and Biotechnology, Zhejiang University, Hangzhou China [12]. It is widely cultivated in southeast China. Seeds were surface sterilized in 0.1% NaClO for 15 min, then rinsed and soaked with distilled water for another 20 min. Seeds were sowed in plastic germination boxes (18 cm × 12 cm × 10 cm) with moistened filter paper. Under dark conditions for two days, germinated seedlings were selected and cultured in a growth chamber with day/night temperatures of 25/20 °C, a 14 h photoperiod, irradiance of 300 µmol m^−2^ s^−1^ and relative humidity of 70%–80%. The nutrient solution was replaced after every five days with Hoagland solution. Samples were divided into three groups including the control group, the group treated with quinclorac, and the group pre-treated with SA under quinclorac stress. According to our previous findings [12], the quinclorac herbicide (0.1 mM) was applied in a solution at four-leaf stage. SA at 10 mg/L was applied in a solution, two days before quinclorac treatment. The treatment concentrations were based on pre-experimental studies (data not shown). After 6 h herbicide exposure, leaf tissues were harvested and frozen in liquid nitrogen for RNA extraction. All materials were stored at −80 °C until further processing.

### 4.2. RNA Isolation and Library Preparation for Transcriptome Analysis

RNA was first extracted and mixed with DNase I to avoid DNA contamination Gill et al. [65]. The oligo (dT) magnetic beads were used to enrich mRNA, and cDNA was synthesized by the fragments of mRNA. After purification with magnetic beads, end reparation and 3′-end single nucleotide A (adenine) addition, sequencing adaptors were ligated to the fragments. Finally, the fragments were amplified by PCR for library construction. The sample library was used for quality and quantity test by Agilent 2100 Bioanaylzer (Agilent, Santa Clara, CA, USA) and ABI StepOnePlus Real-Time PCR System (Applied Biosystems, Foster City, CA, USA). After passing the quality control (QC), the library products were ready for sequencing via Illumina HiSeqTM2000 (Illumina, San Diego, CA, USA).

### 4.3. Analysis of RNA-Sequencing Data

Primary sequencing data was produced by Illumina HiSeqTM 2000, and raw reads were filtered into clean reads. Because raw data may contain low quality reads, adapters and other useless reads, to guarantee the reliability of analysis, the reads containing adapter, poly-N and low quality were removed from the raw data to obtain clean reads. Bowtie2 [66] and BWA [67] were used to map clean reads to reference gene and reference genome, respectively. The alignment with gene/genome reference included genome alignment visualization, map rate statistics, distribution of reads on gene, sequencing saturation and distribution of reads and genes on genome. With good secondary QC, further gene expression analyses were proceeded. RNA-Seq by Expectation-Maximization (RSEM) [68] was applied to use the modeling of the paired-end, the length of reads, fragment length distributions and quality values to distinguish which transcripts were isoforms of the same gene. The fragments per kilobase of transcript per million mapped fragments (FPKM) method [69] can eliminate the influences of gene expression caused by the length of gene or the size of sequencing. By this method, calculated quantification of gene expression level was used to compare the DEG expression level between different samples. NOISeq method [70] was used to screen DEGs between two groups. DEGs were screened according to the following default criteria: Fold change ≥2 and diverge probability ≥0.8.

### 4.4. qRT-PCR Analysis

Total RNA of three different groups were extracted from frozen leaf samples with RNAiso Plus (TaKaRa, Japan). Two micrograms total RNA was subjected to reverse transcription using TaKaRa PrimeScript™ RT reagent Kit with gDNA Eraser (Perfect for Real Time). Real-time PCR was carried out by using SYBRs Premix Ex Taq II (Tli RNaseH Plus) (TaKaRa) in CFX96TM Real-Time System (BIO-RAD, USA). All primers used for qRT-PCR are listed in Table S4.

### 4.5. Protein Extraction

Total leaf proteins were extracted from the same samples for RNA-Sequencing analysis according to Yang et al. [71]. Leaf samples were ground fully in liquid nitrogen and homogenized with 0.1 g of PVPP, 10 mL of Tris-phenol and 1 mL of phenol extraction buffer (with 2% Beta-mercaptoethanol and 1 mM PMSF) at 4 °C. After vortex for 10 s every 5 min with 3 repeats and centrifugation at 6,000 rpm for 20 min, the phenolic phase was collected and precipitated overnight with five volumes of 100 mM ammonium acetate in methanol at −20 °C. The pellet was collected after centrifugation (20 min, 6000 rpm, 4 °C) and suspended in 10 mL of methanol, which was repeated in 10 mL of methanol, 10 mL of acetone and 1 mL of acetone again, respectively. The pellet was collected after centrifugation at 12,000 rpm for 20 min, air-dried and suspended in 150 µL of radioimmunoprecipitation assay (RIPA) lysis buffer containing 0.1% (*v*/*v*) TritonX-100, 1% (*w*/*v*) sodium deoxycholate, 0.1% (*w*/*v*) SDS, 150 mM NaCl and 50 mM Tris-HCl (pH 8.0).

### 4.6. Trypsin Digestion and iTRAQ Labeling

Proteins were digested with trypsin (Promega, Madison, WI, USA) at 37 °C at a ratio of 1:50 (enzyme/substrate) overnight. The iTRAQ labeling was performed according to the manufacturer’s protocol (Applied Biosystems, Sciex, Foster City, CA, USA). All labeled peptides were pooled together.

### 4.7. High-pH Reversed-Phase Chromatography

The Ultimate3000 HPLC system (Dionex, Sunnyvale, CA, USA) equipped with a 2.00-mm-inner diameter × 150-mm-long Gemini-NX 3u C18110A columns (Phenomenex, Torrance, CA, USA) was used for High-pH fractionation. Peptides were loaded onto the column and washed isocratically at 95% eluent A (20 mM HCOONH_4_, 2 M NaOH) (pH 10). Peptide fractionation was performed by using a linear binary gradient from 15% to 50% B (20 mM HCOONH_4_, 2 M NaOH, 80% acetonitrile ACN) (pH 10) at 0.2 mL/min over 45 min. Finally, the column was washed at 90% B for 10 min and returned to 95% A for 10 min. The UV detector was set at 214/280 nm, and fractions were collected every 1 min. In total, 10 fractions were pooled and dried by vacuum centrifuge for subsequent nano-reversed phase liquid chromatography (nano-LC) fractionation.

### 4.8. RPLC-MS/MS Analysis 

Each fraction was suspended in loading buffer (0.1% FA, 2% ACN) and separated using an Ultimate 3000 nano-LC system equipped with a C18 reverse phase column (100-μm inner diameter, 10-cm long, 3-μm resin from Michrom Bioresources, Auburn, CA, USA). The peptides were separated using the following parameters: (1) mobile phase A: 0.1% FA, 5%ACN, dissolved in water; (2) mobile phase B: 0.1% FA, 95% ACN; (3) flow rate: 300 nL/min; (4) gradient: B-phase increased from 5% to 40%, 70 min. Then, the eluent was transferred to Triple TOF 6600 containing multichannel TDC detector with four-anode channel detect ion (AB SCIEX, Concord, ON, Canada). The machine parameters were as follows: 2.5 kv ion source spray voltage, 30 *psi* nitrogen curtain gas, 15 *psi* nebulizer gas, 150 °C interface heater temperature. The mass range was about 400–1250 *m*/*z* in high resolution mode (>30,000) with 250 ms accumulation time per spectrum.

### 4.9. Proteomic Data Analysis

The primary data files (formatted as wiff and wiff. scan) were converted to MGF files using MSConvert and the MGF files were searched. Protein identification was performed by using Mascot search engine (Matrix Science, London, UK; version 2.3.02) against database. The database we selected was Osativa_204 (49061 sequences) with the link http://genome.jgi.doe.gov/pages/dynamicOrganismDownload.jsf?organism = PhytozomeV9.

For protein identification, a mass tolerance of 0.05 Da (50 ppm) was permitted for intact peptide masses and 0.1 Da for fragmented ions, with allowance for one missed cleavages in the trypsin digests. Gln- > pyro-Glu (N-term Q), Oxidation (M), Deamidated (NQ) as the potential variable modifications, and Carbamidomethyl (C), iTRAQ8plex (N-term), iTRAQ8plex (K) as fixed modifications. The charge states of peptides were set to +2 and +3. Specifically, an automatic decoy database search was performed in Mascot by choosing the decoy checkbox in which a random sequence of database is generated and tested for raw spectra as well as the real database. To reduce the probability of false peptide identification, only peptides with significance scores (≥20) at the 99% confidence interval by a Mascot probability analysis greater than “identity” were counted as identified. At least one unique peptide was involved in each confident protein identification.

The peptide data were analyzed using Protein Pilot Software 4.0 (AB SCIEX, Redwood City, CA, USA). Data with a false discovery rate (FDR) of less than 1% were used for the Unused ProtScore measurement. Peptides with scores of over 1.3 (confidence over 95%) were chosen. For quantitative changes, a 2-fold cutoff was set to determine up-regulated and down-regulated. The DEPs were then imported to the clusters of orthologous groups of proteins (COG) database (http://www.ncbi.nlm.nih.gov/COG) for phylogenetic classification and the KEGG database (http://www.genome.jp/kegg/pathway.html) for metabolic pathway analysis.

### 4.10. Correlation Analysis between Transcriptome and Proteome

According to RNA-Sequencing data and proteome analysis, DEGs were identified with the default criteria (Foldchange ≥ 2 and diverge probability ≥ 0.8) by NOISeq method, while DEPs were filtered with the standard (Foldchange ≥ 1.2 and *p* value < 0.05) by protein abundance level. When one protein is expressed in transcriptome level, it is regarded as correlated.

### 4.11. Protein-Protein Interaction Analysis

All identified up-regulated/down-regulated, protein-protein interaction (PPI) was searched against the STRING database (version 10.0) for protein-protein interactions. This database contains interaction from previously published interaction studies as well as genomic analysis established in gene neighborhood, domain fusion and phylogenetic profiling methods. PPIs belong to uploaded data set was selected, whereas confidence score of ≥0.9 was selected to minimize false positive/negative interactions. Stronger associations are represented by thicker lines.

## 5. Conclusions

In summary, we have explored and analyzed the transcriptome and proteomics of *Oryza sativa* ssp. *japonica* to identify and annotate transcripts and proteins associated with quinclorac toxicity and its subsequent alleviation by the exogenous application of SA. Our study suggests that multiple pathways are involved in quinclorac-induced toxicity, which resulted in chlorophyll degradation and the accumulation of endogenous aldehyde, glycolate and other reactive oxygen molecules such as H_2_O_2_ to affect the redox homeostasis. Genes and proteins involved in the crucial steps of chlorophyll synthesis pathways were significantly repressed under quinclorac stress. However, pre-treatment of SA not only modulated plant defense systems but also triggered detoxifying enzymes such as GSH, ALDH, GLO to degrade herbicide or eliminate xenobiotics. Additionally, SA application maintained the chlorophyll content in rice leaves by preventing chlorophyll breakdown and simultaneously accelerating its de novo synthesis. These findings will contribute to an increased understanding of the SA-mediated stress tolerance in rice and also provide experimental data for the development of herbicide resistance in rice breeding programs.

## Figures and Tables

**Table 1:** Summary of average sequencing data for each group.

Group	Total Reads	Quality Filtered Reads	Uniquely Mapped Reads	Genome Mapped Reads (%)	Gene Mapped Reads (%)
Control	13,127,074	13,062,738	11,301,931	86.52	88.71
Q	13,126,991	13,047,776	11,277,174	86.43	89.12
Q + SA	13,127,032	13,028,062	11,223,009	86.15	89.83
Average	13,127,032	13,046,192	11,267,371	86.37	89.22

Clean Data Rate (%) = Clean Reads Number/Raw Reads Number.

**Table 2:** The significant enrichment pathways with differential expressed proteins.

Comparison	Pathway	Differential Proteins with Pathway Annotation (121)	All Proteins with Pathway Annotation (1871)	*p* Value	Pathway ID
Q vs. control	Cysteine and methionine metabolism	9 (7.44%)	39 (2.08%)	0.0005958267	ko00270
	Glyoxylate and dicarboxylate metabolism	8 (6.61%)	47 (2.51%)	0.009008489	ko00630
	Phenylpropanoid biosynthesis	9 (7.44%)	58 (3.1%)	0.01050140	ko00940
	Carbon metabolism	18 (14.88%)	159 (8.5%)	0.01124338	ko01200
	C5-Branched dibasic acid metabolism	2 (1.65%)	5 (0.27%)	0.03646180	ko00660
	Ascorbate and aldarate metabolism	4 (3.31%)	22 (1.18%)	0.04913111	ko00053
	Metabolic pathways	55 (45.45%)	710 (37.95%)	0.04918179	ko01100
Q + SA vs. Q	Alanine, aspartate and glutamate metabolism	5 (5.15%)	28 (1.5%)	0.01281717	ko00250
	Riboflavin metabolism	3 (3.09%)	11 (0.59%)	0.01644878	ko00740
	Arginine biosynthesis	4 (4.12%)	21 (1.12%)	0.02056896	ko00220
	Terpenoid backbone biosynthesis	3 (3.09%)	12 (0.64%)	0.02111914	ko00900
	2-Oxocarboxylic acid metabolism	5 (5.15%)	32 (1.71%)	0.02229215	ko01210
Q + SA vs. control	Photosynthesis	11 (6.29%)	52 (2.78%)	0.006954811	ko00195
	Metabolic pathways	82 (46.86%)	710 (37.95%)	0.007189333	ko01100
	Photosynthesis-antenna proteins	4 (2.29%)	13 (0.69%)	0.02696767	ko00196
	Biosynthesis of amino acids	18 (10.29%)	123 (6.57%)	0.03282929	ko01230
	Pyrimidine metabolism	6 (3.43%)	27 (1.44%)	0.03444194	ko00240
	Valine, leucine and isoleucine biosynthesis	3 (1.71%)	9 (0.48%)	0.04427594	ko00290
	Pentose and glucuronate interconversions	4 (2.29%)	15 (0.8%)	0.04442334	ko00040

**Table 3:** Correlation analysis of transcriptomic and proteomic data under different treatments.

Comparison	Type	Proteins Number	Genes Number	Correlations Number
Q vs. control	Identification	2300	27,342	2202
	Differ expressed	147	2207	44
Q vs. Q+SA	Identification	2300	27,342	2205
	Differ expressed	124	697	5
Q+SA vs. control	Identification	2300	27,342	2205
	Differ expressed	202	1120	35

## Supplementary Materials

Supplementary materials can be found at www.mdpi.com/1422-0067/18/9/1975/s1.
