# Supplementary material for: Complementary RNA-Sequencing Based Transcriptomics and iTRAQ Proteomics Reveal the Mechanism of the Alleviation of Quinclorac Stress by Salicylic Acid in Oryza sativa ssp. japonica
Source: Int J Mol Sci. 2017 Sep 14;18(9):1975. doi: 10.3390/ijms18091975 (PMC5618624; doi:10.3390/ijms18091975)
Supplement: Supplementary file 1 [file ijms-18-01975-s001.pdf]

Supplementary Materials: Complementary RNA-Sequencing Transcriptomics and iTRAQ Proteomics Reveal Mechanism of the Alleviation of Quinclorac Stress by Salicylic Acid in *Oryza sativa* ssp. *Japonica*

Jian Wang [1], Faisal Islam [1], Lan Li [1], Meijuan Long [1], Chong Yang [1], Xiaoli Jin [1], Basharat Ali [1,2], Bizeng Mao [3,*] and Weijun Zhou [1,*]

Table S1.1. The annotation pathways of DEGs in Q vs. control.

No.	Pathway	DEGs with Pathway Annotation (1289)	All Genes with Pathway Annotation (16811)	*p* Value	Pathway ID
1	Glutathione metabolism	46 (3.57%)	139 (0.83%)	3.18E-18	ko00480
2	Porphyrin and chlorophyll metabolism	27 (2.09%)	81 (0.48%)	2.38E-11	ko00860
3	Biosynthesis of secondary metabolites	260 (20.17%)	2346 (13.96%)	1.06E-10	ko01110
4	Metabolic pathways	391 (30.33%)	3931 (23.38%)	1.43E-09	ko01100
5	Valine, leucine and isoleucine degradation	20 (1.55%)	60 (0.36%)	9.16E-09	ko00280
6	Proteasome	24 (1.86%)	84 (0.5%)	1.00E-08	ko03050
7	Glyoxylate and dicarboxylate metabolism	21 (1.63%)	86 (0.51%)	1.47E-06	ko00630
8	Carbon fixation in photosynthetic organisms	23 (1.78%)	101 (0.6%)	1.76E-06	ko00710
9	Protein export	18 (1.4%)	75 (0.45%)	1.06E-05	ko03060
10	Pyruvate metabolism	22 (1.71%)	114 (0.68%)	4.72E-05	ko00620
11	Nitrogen metabolism	14 (1.09%)	65 (0.39%)	0.000337984	ko00910
12	Synthesis and degradation of ketone bodies	5 (0.39%)	10 (0.06%)	0.000477858	ko00072
13	Fatty acid metabolism	12 (0.93%)	53 (0.32%)	0.000547383	ko00071
14	Ubiquinone and other terpenoid-quinone biosynthesis	13 (1.01%)	62 (0.37%)	0.000715332	ko00130
15	Arginine and proline metabolism	20 (1.55%)	122 (0.73%)	0.000954194	ko00330
16	Fructose and mannose metabolism	18 (1.4%)	105 (0.62%)	0.000989257	ko00051
17	Tyrosine metabolism	14 (1.09%)	75 (0.45%)	0.00151435	ko00350
18	Aminoacyl-tRNA biosynthesis	16 (1.24%)	93 (0.55%)	0.001776732	ko00970
19	Vitamin B6 metabolism	7 (0.54%)	25 (0.15%)	0.002154698	ko00750
20	Riboflavin metabolism	9 (0.7%)	41 (0.24%)	0.003314948	ko00740

21	Biosynthesis of unsaturated fatty acids	12 (0.93%)	65 (0.39%)	0.003526822	ko01040
22	Glycolysis/Gluconeogenesis	25 (1.94%)	186 (1.11%)	0.004244406	ko00010
23	Protein processing in endoplasmic reticulum	48 (3.72%)	428 (2.55%)	0.005045713	ko04141
24	Butanoate metabolism	9 (0.7%)	44 (0.26%)	0.00545386	ko00650
25	Pantothenate and CoA biosynthesis	9 (0.7%)	45 (0.27%)	0.006363833	ko00770
26	Selenocompound metabolism	7 (0.54%)	30 (0.18%)	0.006516848	ko00450
27	Pentose phosphate pathway	12 (0.93%)	70 (0.42%)	0.006547296	ko00030
28	Photosynthesis	20 (1.55%)	145 (0.86%)	0.007441267	ko00195
29	Photosynthesis-antenna proteins	5 (0.39%)	17 (0.1%)	0.00746027	ko00196
30	Arachidonic acid metabolism	6 (0.47%)	25 (0.15%)	0.01002171	ko00590
31	Zeatin biosynthesis	22 (1.71%)	172 (1.02%)	0.01228708	ko00908
32	Propanoate metabolism	9 (0.7%)	50 (0.3%)	0.01276795	ko00640
33	Phenylpropanoid biosynthesis	48 (3.72%)	456 (2.71%)	0.0155656	ko00940
34	Flavonoid biosynthesis	31 (2.4%)	276 (1.64%)	0.02071764	ko00941
35	Peroxisome	16 (1.24%)	121 (0.72%)	0.02246085	ko04146
36	Lysine biosynthesis	5 (0.39%)	22 (0.13%)	0.02307562	ko00300
37	Brassinosteroid biosynthesis	10 (0.78%)	66 (0.39%)	0.02802715	ko00905
38	Glycine, serine and threonine metabolism	13 (1.01%)	97 (0.58%)	0.03377495	ko00260
39	Ascorbate and aldarate metabolism	13 (1.01%)	98 (0.58%)	0.0363175	ko00053
40	Phenylalanine metabolism	25 (1.94%)	229 (1.36%)	0.04658659	ko00360

Table S1.2. The annotation pathways of DEGs in Q + SA vs. Q.

No.	Pathway	DEGs with Pathway Annotation (407)	All Genes with Pathway Annotation (16811)	*p* Value	Pathway ID
1	Glutathione metabolism	27 (6.63%)	139 (0.83%)	3.71E-17	ko00480
2	Biosynthesis of secondary metabolites	97 (23.83%)	2346 (13.96%)	4.60E-08	ko01110
3	Phenylpropanoid biosynthesis	30 (7.37%)	456 (2.71%)	7.68E-07	ko00940
4	Tyrosine metabolism	10 (2.46%)	75 (0.45%)	1.26E-05	ko00350
5	Flavonoid biosynthesis	20 (4.91%)	276 (1.64%)	1.38E-05	ko00941
6	Fatty acid metabolism	8 (1.97%)	53 (0.32%)	3.76E-05	ko00071
7	Glycolysis / Gluconeogenesis	15 (3.69%)	186 (1.11%)	4.87E-05	ko00010
8	Metabolic pathways	129 (31.7%)	3931 (23.38%)	6.47E-05	ko01100
9	Valine, leucine and isoleucine degradation	8 (1.97%)	60 (0.36%)	9.37E-05	ko00280
10	Phenylalanine metabolism	16 (3.93%)	229 (1.36%)	0.000151	ko00360
11	Porphyrin and chlorophyll metabolism	8 (1.97%)	81 (0.48%)	0.000758	ko00860
12	Glyoxylate and dicarboxylate metabolism	8 (1.97%)	86 (0.51%)	0.001126	ko00630
13	Carbon fixation in photosynthetic organisms	8 (1.97%)	101 (0.6%)	0.003137	ko00710
14	Zeatin biosynthesis	11 (2.7%)	172 (1.02%)	0.003182	ko00908
15	Ubiquinone and other terpenoid-quinone biosynthesis	6 (1.47%)	62 (0.37%)	0.003804	ko00130
16	Biosynthesis of unsaturated fatty acids	6 (1.47%)	65 (0.39%)	0.004811	ko01040
17	Nitrogen metabolism	6 (1.47%)	65 (0.39%)	0.004811	ko00910
18	alpha-Linolenic acid metabolism	9 (2.21%)	134 (0.8%)	0.005347	ko00592
19	Pyruvate metabolism	8 (1.97%)	114 (0.68%)	0.006523	ko00620
20	Propanoate metabolism	5 (1.23%)	50 (0.3%)	0.007032	ko00640
21	Arginine and proline metabolism	8 (1.97%)	122 (0.73%)	0.009674	ko00330
22	Fructose and mannose metabolism	7 (1.72%)	105 (0.62%)	0.013865	ko00051
23	Cutin, suberine and wax biosynthesis	9 (2.21%)	159 (0.95%)	0.015464	ko00073
24	Stilbenoid, diarylheptanoid and gingerol biosynthesis	14 (3.44%)	304 (1.81%)	0.016548	ko00945
25	beta-Alanine metabolism	4 (0.98%)	45 (0.27%)	0.023104	ko00410

26	Pentose phosphate pathway	5 (1.23%)	70 (0.42%)	0.027216	ko00030
27	Lysine degradation	4 (0.98%)	48 (0.29%)	0.028529	ko00310
28	Glycine, serine and threonine metabolism	6 (1.47%)	97 (0.58%)	0.030396	ko00260
29	Cysteine and methionine metabolism	7 (1.72%)	129 (0.77%)	0.037502	ko00270
30	Anthocyanin biosynthesis	2 (0.49%)	14 (0.08%)	0.043909	ko00942
31	Galactose metabolism	6 (1.47%)	109 (0.65%)	0.049175	ko00052

Table S1.3. The annotation pathways of DEGs in Q+SA vs. control.

No.	Pathway	DEGs with Pathway Annotation (649)	All Genes with Pathway Annotation (16,811)	*p* Value	Pathway ID
1	Ribosome	56 (8.63%)	418 (2.49%)	3.01E-16	ko03010
2	Porphyrin and chlorophyll metabolism	19 (2.93%)	81 (0.48%)	1.74E-10	ko00860
3	Carbon fixation in photosynthetic organisms	14 (2.16%)	101 (0.6%)	3.27E-05	ko00710
4	Photosynthesis	17 (2.62%)	145 (0.86%)	4.49E-05	ko00195
5	Aminoacyl-tRNA biosynthesis	12 (1.85%)	93 (0.55%)	0.000236	ko00970
6	Ubiquinone and other terpenoid-quinone biosynthesis	9 (1.39%)	62 (0.37%)	0.000586	ko00130
7	Biosynthesis of secondary metabolites	119 (18.34%)	2346 (13.96%)	0.0009	ko01110
8	Glyoxylate and dicarboxylate metabolism	10 (1.54%)	86 (0.51%)	0.001729	ko00630
9	Glutathione metabolism	13 (2%)	139 (0.83%)	0.002835	ko00480
10	Nitrogen metabolism	8 (1.23%)	65 (0.39%)	0.003426	ko00910
11	Flavonoid biosynthesis	20 (3.08%)	276 (1.64%)	0.00534	ko00941
12	Protein export	8 (1.23%)	75 (0.45%)	0.008195	ko03060
13	Phenylalanine metabolism	16 (2.47%)	229 (1.36%)	0.016373	ko00360
14	Selenocompound metabolism	4 (0.62%)	30 (0.18%)	0.027194	ko00450
15	Regulation of autophagy	8 (1.23%)	94 (0.56%)	0.02879	ko04140
16	Pantothenate and CoA biosynthesis	5 (0.77%)	45 (0.27%)	0.028902	ko00770

Table S2.1. Transcription factor analysis result in Q vs. control.

Gene ID	TF Family	Up-Down-Regulation (Q/control)	Relative Express Level (Q/control)
LOC_Os05g25260	AP2-EREBP	Up	4.43
LOC_Os08g31580	AP2-EREBP	Up	3.13
LOC_Os01g07120	AP2-EREBP	Up	5.16
LOC_Os04g57340	AP2-EREBP	Up	3.41
LOC_Os01g54890	AP2-EREBP	Up	16.35
LOC_Os02g54160	AP2-EREBP	Up	2.81
LOC_Os02g49450	BSD	Up	2.99
LOC_Os01g46970	bZIP	Up	8.14
LOC_Os08g36790	bZIP	Up	3.55
LOC_Os09g13575	bZIP	Up	2.82
LOC_Os02g49880	C2C2-CO-like	Down	2.45
LOC_Os08g15050	C2C2-CO-like	Up	3.12
LOC_Os06g15330	C2C2-CO-like	Down	3.24
LOC_Os01g24070	C2C2-GATA	Down	2.63
LOC_Os05g06340	C2C2-GATA	Down	4.93
LOC_Os06g37450	C2C2-GATA	Down	3.27
LOC_Os08g03310	C3H	Down	9.76
LOC_Os07g36170	GRAS	Up	2.91
LOC_Os08g40430	mTERF	Down	5.38
LOC_Os07g02800	MYB	Down	4.04
LOC_Os02g04640	MYB	Up	10.31
LOC_Os03g55590	MYB	Down	4.78
LOC_Os11g47460	MYB-related	Down	9.69
LOC_Os01g44390	MYB-related	Down	3.42
LOC_Os02g42850	MYB-related	Up	7.58
LOC_Os01g04930	MYB-related	Up	3.48
LOC_Os01g03660	MYB-related	Down	14.67
LOC_Os09g01960	MYB-related	Up	5.03
LOC_Os01g47370	MYB-related	Down	14.67
LOC_Os11g03300	NAC	Up	62.64
LOC_Os07g48550	NAC	Up	8.17
LOC_Os12g03040	NAC	Up	125.00
LOC_Os10g42410	PLATZ	Up	3.18
LOC_Os11g26160	Sigma70-like	Down	5.97
LOC_Os07g42370	Tify	Up	3.99
LOC_Os03g28940	Tify	Up	9.36
LOC_Os05g27730	WRKY	Up	2.58
LOC_Os12g40570	WRKY	Up	3.23
LOC_Os02g08440	WRKY	Up	3.95
LOC_Os03g55164	WRKY	Down	5.70
LOC_Os01g14440	WRKY	Up	8.26

Table S2.2. Transcription factor analysis result in Q + SA vs. Q.

Gene ID	TF Family	Up-Down-Regulation (Q + SA/Q)	Relative Express Level (Q + SA/Q)
LOC_Os04g57340	AP2-EREBP	Down	2.58
LOC_Os01g54890	AP2-EREBP	Down	3.90
LOC_Os01g72370	bHLH	Up	29.97
LOC_Os06g15330	C2C2-CO-like	Up	3.24
LOC_Os08g03310	C3H	Up	3.92
LOC_Os02g04640	MYB	Down	4.36
LOC_Os11g47460	MYB-related	Up	15.57
LOC_Os06g51070	NAC	Up	6.42
LOC_Os08g42400	NAC	Down	2.38
LOC_Os11g03300	NAC	Down	8.44
LOC_Os12g03040	NAC	Down	4.46
LOC_Os04g40630	TAZ	Up	2.89
LOC_Os03g28940	Tify	Down	2.39
LOC_Os05g25770	WRKY	Up	5.75
LOC_Os09g25060	WRKY	Up	10.82

Table S2.3. Transcription factor analysis result in Q + SA vs. control.

Gene ID	TF Family	Up-Down-Regulation (Q + SA/control)	Relative Express Level (Q + SA/control)
LOC_Os05g25260	AP2-EREBP	Up	2.77
LOC_Os01g54890	AP2-EREBP	Up	4.19
LOC_Os01g72370	bHLH	Up	115.87
LOC_Os03g53020	bHLH	Up	4.31
LOC_Os04g23440	bHLH	Up	3.26
LOC_Os09g13575	bZIP	Up	3.68
LOC_Os08g15050	C2C2-CO-like	Up	3.18
LOC_Os03g55540	C2H2	Up	2.66
LOC_Os08g03310	C3H	Down	2.49
LOC_Os08g40430	mTERF	Down	4.48
LOC_Os08g33750	MYB	Up	3.27
LOC_Os03g55590	MYB	Down	4.81
LOC_Os01g44390	MYB-related	Down	3.65
LOC_Os01g03660	MYB-related	Down	5.16
LOC_Os01g47370	MYB-related	Down	506.00
LOC_Os12g37970	MYB-related	Up	2.69
LOC_Os06g51070	NAC	Up	4.88
LOC_Os07g48550	NAC	Up	7.13
LOC_Os03g21060	NAC	Up	4.46
LOC_Os12g03040	NAC	Up	28.02
LOC_Os10g42410	PLATZ	Up	2.62
LOC_Os11g26160	Sigma70-like	Down	5.44
LOC_Os03g08330	Tify	Up	8.63
LOC_Os07g42370	Tify	Up	3.82
LOC_Os03g28940	Tify	Up	3.92
LOC_Os03g27080	TIG	Up	3.59
LOC_Os08g29660	WRKY	Up	3.87
LOC_Os05g25770	WRKY	Up	7.68
LOC_Os03g55164	WRKY	Down	7.39
LOC_Os09g25060	WRKY	Up	59.56
LOC_Os09g25070	WRKY	Up	24.27
LOC_Os01g14440	WRKY	Up	4.35

Table S3. Correlated differential expressed proteins and genes with same expression trend.

Comparison	Gene ID and Protein ID	Description	Protein Fold Change	Gene Fold Change	Protein Up or Down	Gene Up or Down
Q vs. control	LOC_Os07g30670.1|PACid:24113474	NDH-dependent cyclic electron flow 1	0.793	−4.03749	−	−
	LOC_Os03g20700.1|PACid:24121916	magnesium-chelatase subunit chlH, chloroplast, putative/Mg-protoporphyrin IX chelatase, putative	0.726	−1.79826	−	−
	LOC_Os12g34890.1|PACid:24147998	acyl carrier protein 4	0.729	−2.52412	−	−
	LOC_Os02g02400.1|PACid:24131177	catalase 2	0.618	−3.17371	−	−
	LOC_Os04g23820.1|PACid:24106046	Nucleic acid-binding, OB-fold-like protein	0.802	−1.50192	−	−
	LOC_Os06g20320.1|PACid:24140328	trigger factor type chaperone family protein	0.752	−1.90953	−	−
	LOC_Os02g51080.1|PACid:24130840	Pyridine nucleotide-disulphide oxidoreductase family protein	0.786	−1.10899	−	−
	LOC_Os03g53860.2|PACid:24126462	Glycosyl hydrolase family protein	0.776	−2.93984	−	−
	LOC_Os05g22614.1|PACid:24153327	plastid transcriptionally active 16	0.766	−1.62716	−	−
	LOC_Os03g34040.1|PACid:24125760	Ribosomal protein S5 family protein	0.652	−2.13044	−	−

LOC_Os03g20300.1|PACid:24121754	glucose-6-phosphate dehydrogenase 4	1.542	1.955432	+	+
LOC_Os02g09150.1|PACid:24130420	Inorganic H pyrophosphatase family protein	0.757	−1.61497	−	−
LOC_Os10g35370.1|PACid:24096134	protochlorophyllide oxidoreductase A	0.83	−1.68683	−	−
LOC_Os02g39870.1|PACid:24130496	Co-chaperone GrpE family protein	0.729	−1.57108	−	−
LOC_Os04g59150.1|PACid:24104476	Peroxidase superfamily protein	0.682	−3.55338	−	−
LOC_Os08g23150.1|PACid:24101120	Aldolase-type TIM barrel family protein	1.719	2.139437	+	+
LOC_Os11g05110.1|PACid:24158078	Pyruvate kinase family protein	1.332	1.562643	+	+
LOC_Os01g13690.2|PACid:24121217	D-aminoacid aminotransferase-like PLP-dependent enzymes superfamily protein	0.705	−2.80709	−	−
LOC_Os04g50110.1|PACid:24103562	RNA-binding (RRM/RBD/RNP motifs) family protein	0.698	−2.74162	−	−
LOC_Os05g32220.1|PACid:24152509	Ribosomal protein L1p/L10e family	0.788	−1.13545	−	−
LOC_Os01g01120.1|PACid:24115157	haloacid dehalogenase-like hydrolase family protein	0.521	−1.6037	−	−
LOC_Os03g10060.1|PACid:24125633	Ribosomal protein S10p/S20e family protein	0.681	−1.38938	−	−
LOC_Os06g21530.1|PACid:24141728	Protein of unknown function (DUF3464)	0.685	−1.78034	−	−

LOC_Os12g43630.1|PACid:24149304	peroxisomal NAD-malate dehydrogenase 1	2.04	1.831354	+	+
LOC_Os05g47540.1|PACid:24154801	S-adenosyl-L-methionine-dependent methyltransferases superfamily protein	0.741	−5.01774	−	−
LOC_Os06g22690.1|PACid:24143762	DnaJ/Hsp40 cysteine-rich domain superfamily protein	0.771	−2.57946	−	−
LOC_Os06g13660.1|PACid:24141257	Alanyl-tRNA synthetase, class IIc	0.786	−1.21978	−	−
LOC_Os02g51570.1|PACid:24134513	FK506-binding protein 16-2	0.729	−2.95299	−	−
LOC_Os09g38090.1|PACid:24135446	plastid movement impaired1	0.804	−1.42739	−	−
LOC_Os07g06450.1|PACid:24113121	chloroplast RNA-binding protein 33	0.616	−3.28249	−	−
LOC_Os02g35630.1|PACid:24135287	ATP-dependent Clp protease	0.289	1.383269	−	+
LOC_Os02g37060.1|PACid:24135031	Photosystem II 5 kD protein	1.443	−1.27741	+	−
LOC_Os05g27100.1|PACid:24149810	Heavy metal transport/detoxification superfamily protein	1.557	−3.6248	+	−
LOC_Os06g04270.1|PACid:24139290	Transketolase	1.269	−2.45628	+	−
LOC_Os03g17580.1|PACid:24125193	Ribosomal protein L10 family protein	1.552	−1.70679	+	−
LOC_Os07g11110.1|PACid:24109294	chloroplast stem-loop binding protein of 41 kDa	1.299	−1.57746	+	−

	LOC_Os04g26910.1|PACid:24103370	NAD(P)-linked oxidoreductase superfamily protein	0.732	1.463961	−	+
	LOC_Os01g13210.1|PACid:24115330	plasma-membrane associated cation-binding protein 1	0.82	2.05599	−	+
	LOC_Os04g55720.1|PACid:24102974	D-3-phosphoglycerate dehydrogenase	0.731	1.8551	−	+
	LOC_Os12g23180.1|PACid:24149046	chloroplast RNA binding	1.272	−1.60368	+	−
	LOC_Os03g40270.1|PACid:24122861	reversibly glycosylated polypeptide 2	0.796	1.494137	−	+
	LOC_Os07g48020.1|PACid:24112080	Peroxidase superfamily protein	0.707	2.981572	−	+
	LOC_Os05g40010.1|PACid:24152888	Bifunctional inhibitor/lipid-transfer protein/seed storage 2S albumin superfamily protein	0.618	4.133919	−	+
	LOC_Os08g01380.1|PACid:24102239	2Fe-2S ferredoxin-like superfamily protein	1.848	−2.4824	+	−
Q+SA vs. Q	LOC_Os02g41630.2|PACid:24134116	PHE ammonia lyase 1	1.82	2.159383	+	+
	LOC_Os12g01530.1|PACid:24148942	ferritin 4	0.203	−1.18402	−	−
	LOC_Os05g25850.1|PACid:24151755	manganese superoxide dismutase 1	0.802	−1.86968	−	−
	LOC_Os12g08760.1|PACid:24145205	Phosphoenolpyruvate carboxylase family protein	0.667	1.892511	−	+
	LOC_Os12g39360.1|PACid:24145239	Eukaryotic aspartyl protease family protein	0.706	1.8623	−	+

	LOC_Os03g16860.1|PACid:24123810	heat shock protein 70	1.957	1.609228	+	+
	LOC_Os07g30670.1|PACid:24113474	NDH-dependent cyclic electron flow 1	0.781	−2.5813	−	−
	LOC_Os12g34890.1|PACid:24147998	acyl carrier protein 4	0.585	−2.68503	−	−
	LOC_Os01g05060.1|PACid:24120705	Mitochondrial glycoprotein family protein	0.612	−2.24827	−	−
	LOC_Os04g45490.1|PACid:24107977	Translation elongation factor EFG/EF2 protein	0.776	−2.13393	−	−
	LOC_Os06g20320.1|PACid:24140328	trigger factor type chaperone family protein	0.53	−1.86912	−	−
Q+SA vs. control	LOC_Os01g05080.1|PACid:24116677	Tetratricopeptide repeat (TPR)-like superfamily protein	0.744	−1.82578	−	−
	LOC_Os02g39870.1|PACid:24130496	Co-chaperone GrpE family protein	0.637	−1.90948	−	−
	LOC_Os03g21900.1|PACid:24127127	Uroporphyrinogen decarboxylase	0.8	−2.59497	−	−
	LOC_Os03g04470.1|PACid:24122321		0.823	−2.15256	−	−
	LOC_Os12g01530.1|PACid:24148942	ferritin 4	0.289	−1.73803	−	−
	LOC_Os07g28400.1|PACid:24111758	2 iron, 2 sulfur cluster binding	0.259	−1.32899	−	−
	LOC_Os03g14040.1|PACid:24128197	DnaJ/Hsp40 cysteine-rich domain superfamily protein	0.518	−1.65709	−	−

LOC_Os02g22260.1|PACid:24131679	FAD/NAD(P)-binding oxidoreductase	0.788	−1.45154	–	–
LOC_Os10g35810.1|PACid:24096485	Tetratricopeptide repeat (TPR)-like superfamily protein	0.528	−2.53343	–	–
LOC_Os03g36540.1|PACid:24127116	magnesium chelatase i2	0.723	−2.26205	–	–
LOC_Os02g08380.1|PACid:24132791	Uncharacterised BCR, YbaB family COG0718	0.667	−1.84739	–	–
LOC_Os12g14070.1|PACid:24149307	chloroplast heat shock protein 70-2	0.802	−1.31372	–	–
LOC_Os05g09400.1|PACid:24149728	plastid-specific ribosomal protein 4	0.687	−2.34534	–	–
LOC_Os02g04460.1|PACid:24133901	Ribosomal protein L3 family protein	0.579	−2.29711	–	–
LOC_Os04g16770.1|PACid:24103392	photosystem II reaction center protein A	0.702	−4.92418	–	–
LOC_Os02g09590.1|PACid:24135086	Ribosomal protein S21 family protein	0.581	−1.97266	–	–
LOC_Os06g22690.1|PACid:24143762	DnaJ/Hsp40 cysteine-rich domain superfamily protein	0.429	−2.61996	–	–
LOC_Os05g49800.1|PACid:24152882	ketol-acid reductoisomerase	0.696	−2.99062	–	–
LOC_Os02g51570.1|PACid:24134513	FK506-binding protein 16-2	0.777	−2.21022	–	–
LOC_Os08g01380.1|PACid:24102239	2Fe-2S ferredoxin-like superfamily protein	0.801	−1.83672	–	–

LOC_Os07g06450.1|PACid:24113121	chloroplast RNA-binding protein 33	0.348	-2.88365	−	−-
LOC_Os12g36640.1|PACid:24149222	Adenine nucleotide alpha hydrolases-like superfamily protein	0.429	1.594161	−	+
LOC_Os02g47600.1|PACid:24132270	pyrophosphorylase 1	0.334	1.369838	−	+
LOC_Os06g04270.1|PACid:24139290	Transketolase	1.424	-1.27221	+	−
LOC_Os02g41630.2|PACid:24134116	PHE ammonia lyase 1	0.746	1.410664	−	+
LOC_Os08g15460.1|PACid:24099089	SECY homolog 1	1.371	−2.85231	+	−
LOC_Os03g40270.1|PACid:24122861	reversibly glycosylated polypeptide 2	0.833	1.540585	−	+
LOC_Os03g14654.1|PACid:24127856	Bifunctional inhibitor/lipid-transfer protein/seed storage 2S albumin superfamily protein	0.79	1.900674	−	+
LOC_Os11g02440.1|PACid:24156174	Chalcone-flavanone isomerase family protein	0.504	1.725554	−	+

Table S4. Primers used for RT-PCR

Selected gene	Sequence (5'-3')	Selected gene	Sequence (5'-3')
Actin F	ATGCTCTCCCCCATGCTATC	OsALDH7 F	GCACCGGTTGTGAGAGAAGA
Actin R	TCTTCCTTGCTCATCCTGTC	OsALDH7 R	CCATGAGGCCCAATCCACTT
OsGR1 F	TCTCAGAGGGACTTCTCTACT	OsALDH6B2 F	GCGATGTATTCCGTGGGCTA
OsGR1 R	AGGCAGTGGTACTCACATGGT	OsALDH6B2 R	ACCAGCAGCAATAAGGGCAT
OsGR2 F	GTGTACTCTGGTTTGCATCT	Os3BGlu7 F	GGCAATCGTGTCAAGCACTG
OsGR2 R	CTGCAGGCAGAACGAATGAT	Os3BGlu7 R	TCTGTTGCTGAGTTCCCACC
OsGR3 F	CAACAGACAGATATCGGTA	OsBIABP1 F	GAGGCGATCGGCTTCGAGGTC
OsGR3 R	TACTATCAACATCCTGAAGC	OsBIABP1 R	GTGGACGTCTTCGGTAACTCGTC
OsAPX2 F	AGAGTCAGTACGATCAAGAC	OsPAL1 F	AAGGTGTTCCTCGGCATCAG
OsAPX2 R	TCTTGACAGCAAATAGCTTGG	OsPAL1 R	GGCAATGGCGATGGGATCTT
OsAPX7 F	TGAGCCAGATCGCTGAAGTG	OsPAL2 F	GCATCAGCTTCCAACTCG
OsAPX7 R	TCCAATATGACTCGTGGTCA	OsPAL2 R	GGTTTCGCACTCCATTACAGA
OsGSTU4 F	CTACGTCGACGACAAGTTCGTT	OsPAL4 F	CTTCACAACAGCTAATCGAG
OsGSTU4 R	TCCTCCGTCTTGCCTCTGAA	OsPAL4 R	CGCACTCCATTTCAGTACCA
OsALDH2B5 F	AGCAGGGGGTTCTACATCCA	OsSCP46 F	TCAAGTTTGACGTAGCTGGGTA
OsALDH2B5 R	CTCCACCGTGCTGAACTTGA	OsSCP46 R	CTCACGGGCGTAGGAGAAC

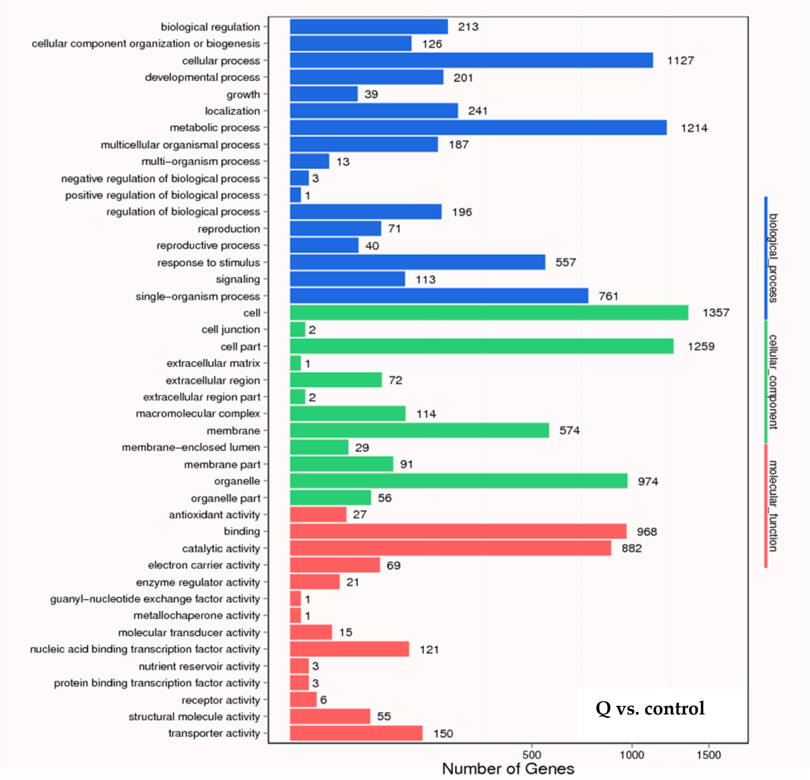
biological regulation
cellular component organization or biogenesis
cellular process
developmental process
growth
localization
metabolic process
multicellular organismal process
multi–organism process
negative regulation of biological process
positive regulation of biological process
regulation of biological process
reproduction
reproductive process
response to stimulus
signaling
single–organism process
cell
cell junction
cell part
extracellular matrix
extracellular region
extracellular region part
macromolecular complex
membrane
membrane–enclosed lumen
membrane part
organelle
organelle part
antioxidant activity
binding
catalytic activity
electron carrier activity
enzyme regulator activity
guanyl–nucleotide exchange factor activity
metallochaperone activity
molecular transducer activity
nucleic acid binding transcription factor activity
nutrient reservoir activity
protein binding transcription factor activity
receptor activity
structural molecule activity
transporter activity
213
126
1127
201
39
241
1214
187
13
3
1
196
71
40
557
113
761
1357
2
1259
1
72
2
114
574
29
91
974
56
27
968
882
69
21
1
1
15
121
3
3
6
55
150
500
1000
1500
Number of Genes
biological_process
cellular_component
molecular_function
Q vs. control

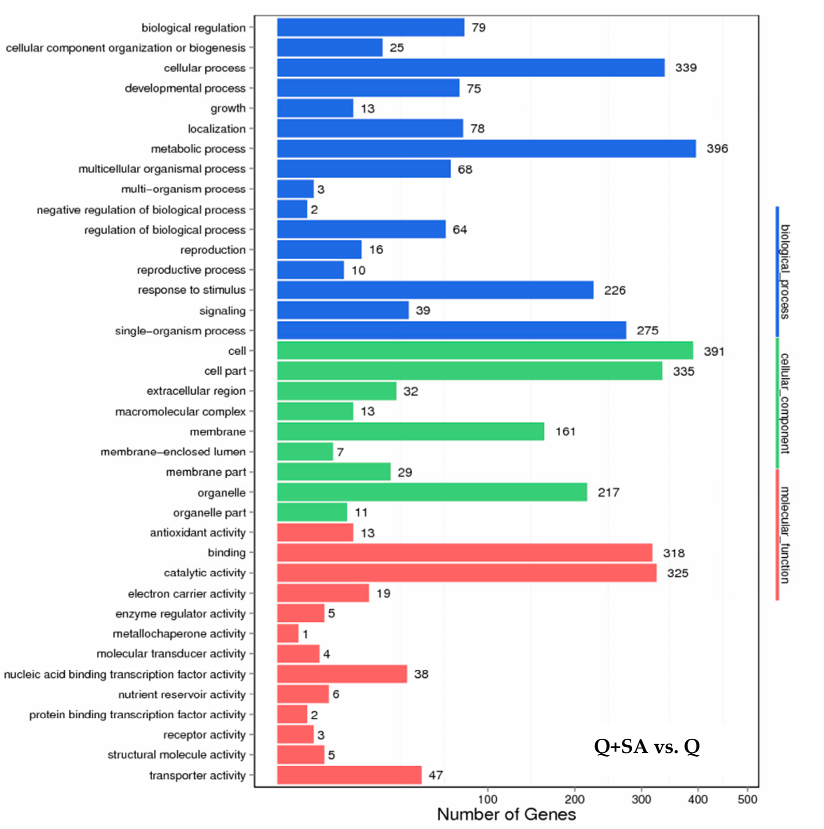
biological regulation
cellular component organization or biogenesis
cellular process
developmental process
growth
localization
metabolic process
multicellular organismal process
multi–organism process
negative regulation of biological process
regulation of biological process
reproduction
reproductive process
response to stimulus
signaling
single–organism process
cell
cell part
extracellular region
macromolecular complex
membrane
membrane–enclosed lumen
membrane part
organelle
organelle part
antioxidant activity
binding
catalytic activity
electron carrier activity
enzyme regulator activity
metallochaperone activity
molecular transducer activity
nucleic acid binding transcription factor activity
nutrient reservoir activity
protein binding transcription factor activity
receptor activity
structural molecule activity
transporter activity
79
25
339
75
13
78
396
68
3
2
64
16
10
226
39
275
391
335
32
13
161
7
29
217
11
13
318
325
19
5
1
4
38
6
2
3
5
47
100
200
300
400
500
Number of Genes
biological_process
cellular_component
molecular_function
Q+SA vs. Q

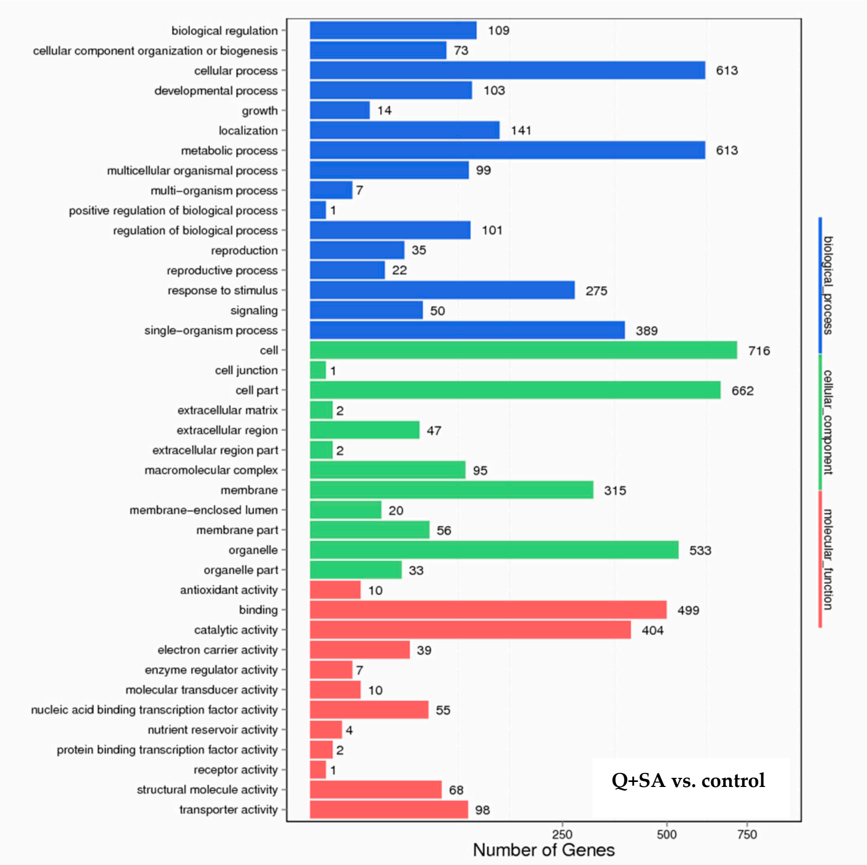

Figure S1. GO functional classification on DEGs for each pairwise. All GO terms are grouped in to three ontologies: blue is for biological process, brown is for cellular component and orange is for molecular function.

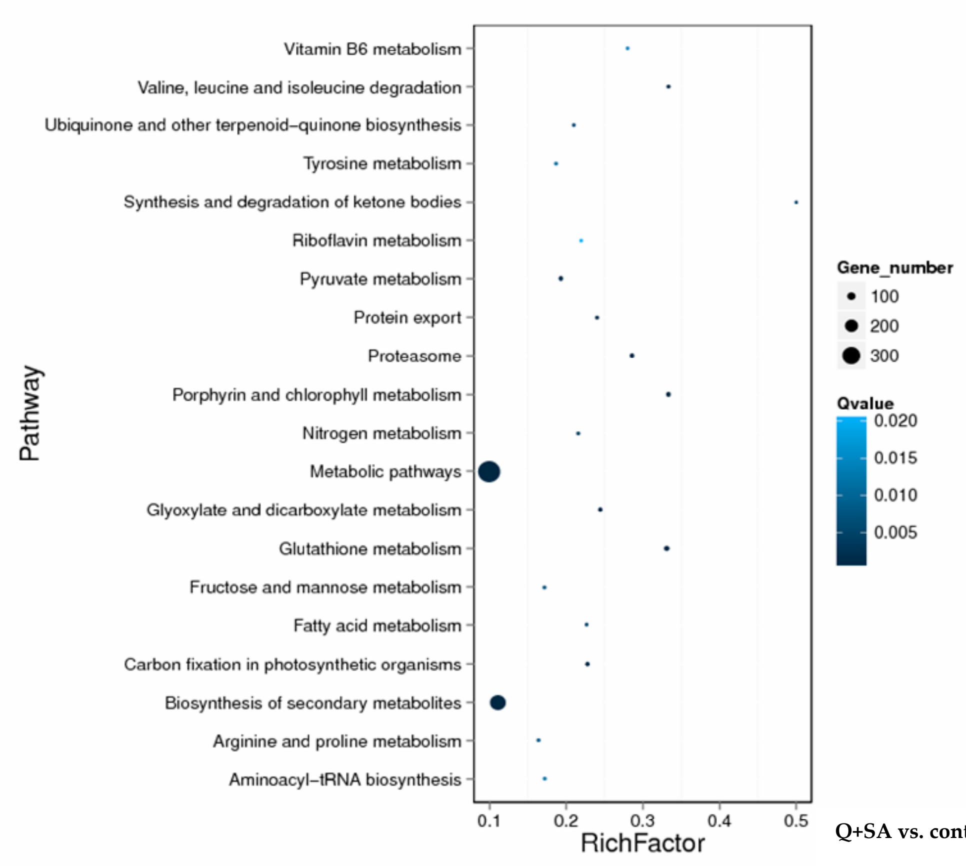

Q+SA vs. control

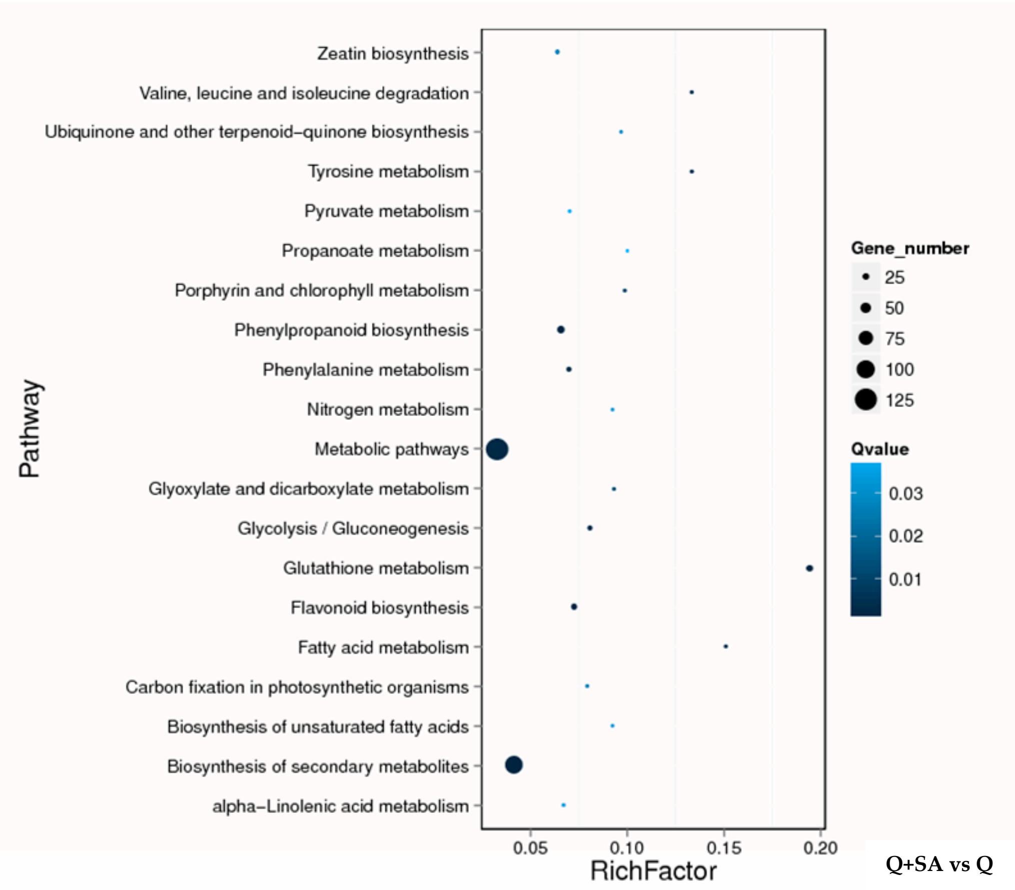

Q+SA vs Q

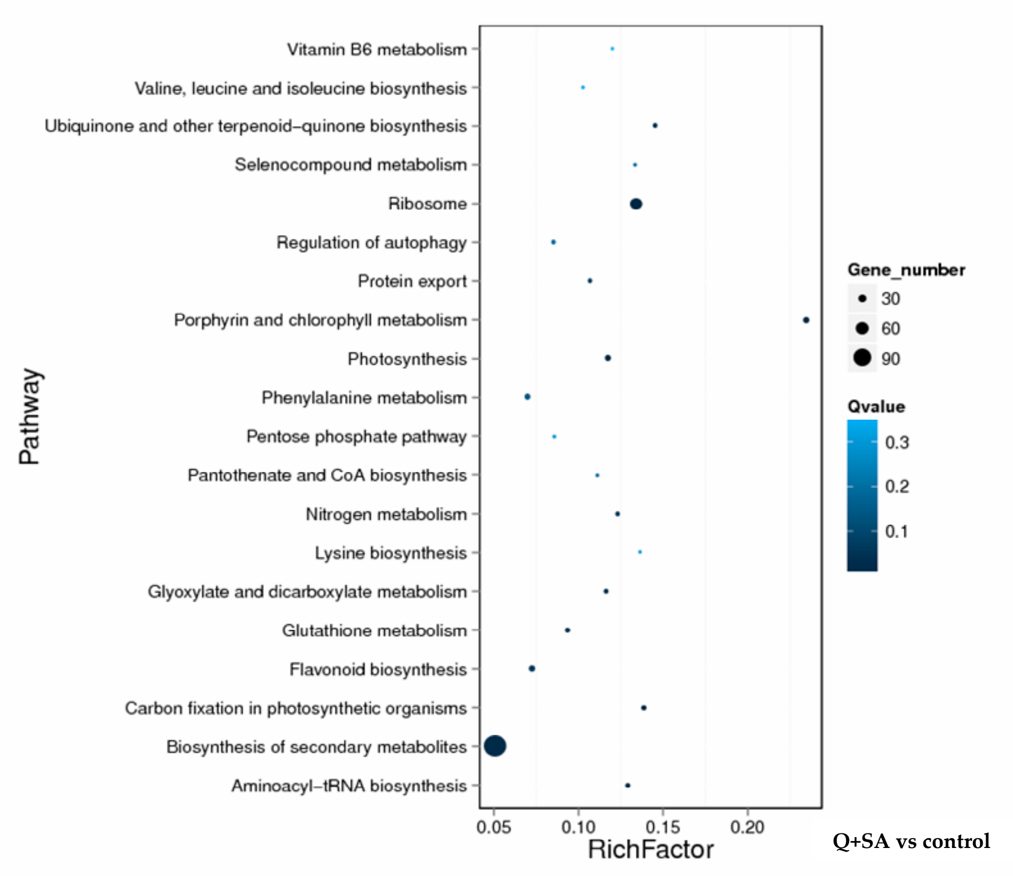

Figure S2. Statistics of top 20 enriched pathway terms in each pairwise. RichFactor is the ratio of differentially expressed gene numbers annotated in this pathway term to all gene numbers annoted in this pathway term. Greater richFator means greater intensiveness. Qvalue is corrected pvalue ranging from 0 ~ 1, and less Qvalue means greater intensiveness.
